# Multifactorial engineering of biomimetic membranes for batteries with multiple high-performance parameters

**DOI:** 10.1038/s41467-021-27861-w

**Published:** 2022-01-12

**Authors:** Mingqiang Wang, Ahmet E. Emre, Ji-Young Kim, Yiting Huang, Li Liu, Volkan Cecen, Yudong Huang, Nicholas A. Kotov

**Affiliations:** 1 School of Chemistry and Chemical Engineering, Harbin Institute of Technology, Harbin, 150001 People’s Republic of China; 2 Department of Chemical Engineering, University of Michigan, Ann Arbor, MI 48109 USA; 3 Biointerfaces Institute, University of Michigan, Ann Arbor, MI 48109 USA; 4 Department of Materials Science and Engineering, University of Michigan, Ann Arbor, MI 48109 USA; 5 Department of Biomedical Engineering, University of Michigan, Ann Arbor, MI 48109 USA

**Keywords:** Batteries, Organic-inorganic nanostructures

## Abstract

Lithium–sulfur (Li–S) batteries have a high specific capacity, but lithium polysulfide (LPS) diffusion and lithium dendrite growth drastically reduce their cycle life. High discharge rates also necessitate their resilience to high temperature. Here we show that biomimetic self-assembled membranes from aramid nanofibers (ANFs) address these challenges. Replicating the fibrous structure of cartilage, multifactorial engineering of ion-selective mechanical, and thermal properties becomes possible. LPS adsorption on ANF surface creates a layer of negative charge on nanoscale pores blocking LPS transport. The batteries using cartilage-like bioinspired ANF membranes exhibited a close-to-theoretical-maximum capacity of 1268 mAh g^−1^, up to 3500+ cycle life, and up to 3C discharge rates. Essential for safety, the high thermal resilience of ANFs enables operation at temperatures up to 80 °C. The simplicity of synthesis and recyclability of ANFs open the door for engineering high-performance materials for numerous energy technologies.

## Abstract (web summary)

Lithium–sulfur batteries have a high specific capacity, but lithium polysulfide diffusion (LPS) and dendrite growth reduce their cycle life. Here, the authors show a biomimetic aramid nanofiber membrane for effectively suppressing LPS diffusion as well as lithium dendrites while allowing lithium ions to be transported. The membranes resists performance degradation at high temperatures and can be produced at scale by Kevlar recycling.

## Introduction

The high theoretical specific capacity of 1675 mAh g^−1^, environmental friendliness, and earth-abundance of elements forming lithium–sulfur (Li–S) batteries make them an attractive platform for energy storage in a variety of technological fields from electric vehicles to robotics and from power grids to aerospace engineering^1^. However, the diffusion of lithium polysulfides (LPS, Li_2_S_*X*_, 4 ≤ *x* ≤ 8)^2^ from cathode to anode drastically reduces their cycle life, overall capacity, and Coulombic efficiency^3,4^. Additionally, LPS layers passivate both the electrodes, leading to a significant increase in impedance and thus to energy losses^5^. The non-uniform surface layer on the anode also promotes the growth of dendrites, which represents another serious issue for Li–S batteries that causes similar issues compounded by short-circuiting and overheating.

The extensive research effort in the past was invested into designing materials for sulfur cathode that would minimize LPS release. It was shown that immobilization of LPS is possible by encapsulating sulfur into microporous carriers made from nanocarbons^6,7^, conductive polymers^8,9^, transition metal oxides^1^, and metal-organic frameworks^10,11^. Indeed, nanoporous barriers in the cathode improved the retention of sulfur within the cathode. However, there is still considerable room for improvement in the cycle life and overall performance of Li–S batteries addressing the structural complexity of cathode material and improving electron transport through electrode material^12,13^.

The problem of the LPS diffusion can also be approached by optimizing the materials design of ion-conducting membranes that can block the LPS transport from S cathode to Li anode. Simultaneously, these membranes must allow the facile transport of Li^+^ ions^5,14^. Significant advances in this area were achieved using coatings from carbonaceous materials^15–17^, polymers^18^, metal foams^19–21^ metal-oxide layers^22,23^, and metal oxides with carbon^24,25^. The great challenge for all of these materials  solutions for LPS membranes is to combine at least two contrarian materials properties—efficient ion transport and mechanical robustness in one material or a coating^5,15,26^. Among the latter, polymers with high shear modulus are necessary to suppress dendrite growth on lithium anodes^27^ while high mechanical strength (>98 MPa) and thermal stability (1 h @ 90C with <5% shrinkage) are essential for the longevity of the batteries in real-world conditions^28^, for instance, in electric vehicles. The prior experimental and computational data show this materials engineering task is difficult^5,15,26,29,18^ and requires a new approach in materials design.

Here, we show that the ion-selective membranes engineered using sequential deposition of nanofibers enable nearly complete prevention of the LPS diffusion from cathode to anode. The structural design for this membrane was informed by the prior studies of ion channels in cell membranes known for efficient ion transport combined with high ion selectivity^30^, as well as cartilage known for unique mechanical properties^22^. The design of cartilage and several other biological tissues is based on highly interconnected nanofiber networks engendering their unique mechanical, adhesive, and transport properties^23,31^. Their topology reflected indistinct interconnectivity, and percolation enables effective stress transfer providing the toughness and flexibility, which can be replicated in composites made from aramid nanofibers (ANFs). ANFs are the nanoscale version of Kevlar fibers^32^, and multifunctional ANF-based composites have been fabricated inspired by cartilage^33^. The direct analogy between the organization of nanofibers in ANF membranes and cartilage was recently demonstrated by evaluating their connectivity using Graph Theory^34^. ANF-based composites can also be engineered into stratified membranes with nanoscale porosity (*np-ANF*) and charge sieving capabilities due to the spontaneous adsorption of LPS layer on *np-ANF* surface. Numerical simulations confirm that negatively charged single-nanometer pores of *np-ANF* strongly inhibit LPS shuttling while affording rapid transport of Li^+^ ions.

ANF-based composites have been utilized as electrode materials^35,36^ and separators^37–39^ in various energy storage systems^40,41^ including lithium sulfur batteries^42^ due to their high mechanical and thermal properties, similarly, *np-ANF* membranes also display a high Young’s modulus of *E* = 9.2 ± 0.5 GPa (55 times higher than *Celgard*^*TM*^ 2400) and high thermal resistance (600 °C). These properties make possible simultaneous suppression of lithium dendrites extending the life cycle of the Li–S batteries to 3500+ cycles at 3C. The combination of properties found in *np-ANF* resulted in high efficiency of LPS blocking and remarkable stability over long-term cycling, even for high-temperature environments.

## Results

### Characterization of the *np-ANF* membrane

**Fig. 1:**
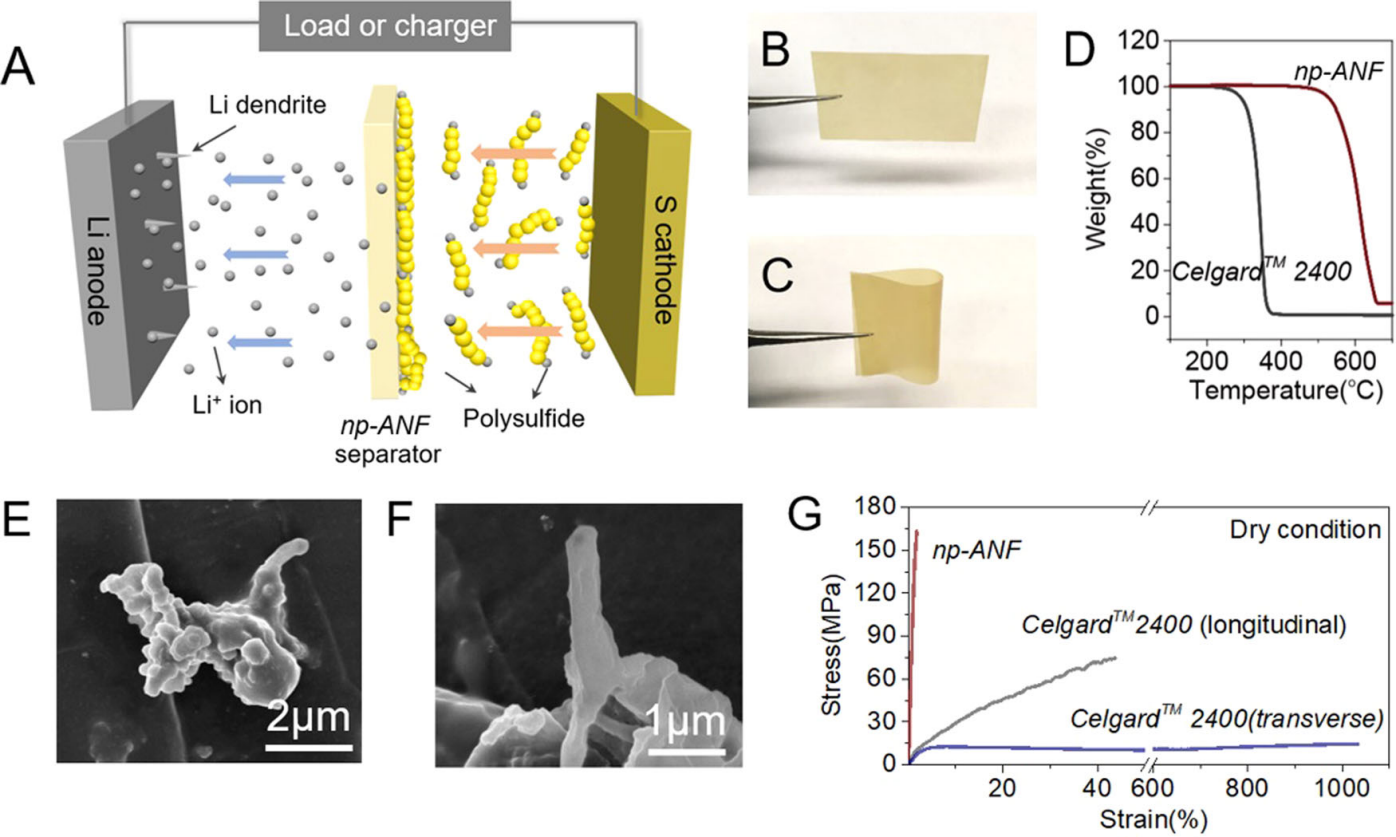
Characterization of the *np-ANF* membrane. **A** Schematic configuration of a Li–S cell with a *np-ANF* membrane between the sulfur cathode and the lithium anode. **B**, **C** Photographs of an *np-ANF* membrane. **D** Thermogravimetric analysis curves for *np-ANF* membrane and *Celgard*^*TM*^ 2400. **E**, **F** SEM images of the tip of lithium dendrite. **G** Stress–strain curves for *np-ANF* and *Celgard*^*TM*^ 2400.

A method of spin-assisted layering assembly^43^ with a positively charged poly(diallyldimethylammonium) partner polymer (PDDA) facilitating adhesion of the subsequent spin-layers is conceptually similar layer-by-layer (LBL) deposition^44,45^ was used to fabricate membranes from ANF dispersions (see “Methods”). Using this technique, the thickness of membrane can be easily varied by increasing or decreasing the number of deposition cycles (Supplementary Fig. 1), which is required for the optimization of flux and selectivity of the ion-transporting membranes. By repeating the spin deposition cycle three times, we obtained ANF composite membranes with a smooth surface (Fig. 1B, C and Supplementary Fig. 1) and a thickness of 5.8 ± 0.50 µm (Supplementary Fig. 1D), which is much about four times thinner than that of *Celgard*^*TM*^ 2400 membranes with a typical thickness of 25 µm. Reducing the membrane thickness represents a critical technological target because it enables increasing the thickness of active materials on both cathode and anode, increasing the overall battery capacity. The thickness of the state-of-the-art ion-conducting membranes for Li–S batteries is between 20 and 30 µm. (Supplementary Tables 5–8). Thermogravimetric analysis (TGA) on *np-ANF* membrane indicates that no significant weight loss occurs below 600 °C in N_2_ atmosphere (Fig. 1D), and the differential scanning calorimetry (DSC) analysis on *np-ANF* confirms no significant change occurring with *np-ANF* until 500 °C (Supplementary Figs. 2, 3). *np-ANF* membranes also show lower electrolyte contact angle and higher electrolyte uptake compared to *Celgard*^*TM*^ *2400*, which reduces internal resistance needed to achieve superior rate charge−discharge rate (Supplementary Fig. 4). The highly interconnected cartilage-like pectolated network of nanofibers also engenders small volume changes of *np-ANF* after being fully wetted by electrolyte, which facilitates tight adhesion to electrodes and is needed for efficient dendrite suppression that may not be the case for many other membrane architectures (Supplementary Fig. 5). *np-ANF* membranes obtained by spin-coating with PDDA also features nanoscale pores 0.9–1.2 nm in diameter as determined by the Barrett-Joyner-Halenda (BJH) analysis (Supplementary Fig. 6), which is smaller than the hydrodynamic diameter of L_2_S_4_ of 1.3–1.5 nm^2,46,47^. These pores are at least one order of magnitude smaller than those found in *Celgard*^*TM*^ 2400 where the BJH pore size exceeds 25 nm (Supplementary Fig. 7).

### Synergistic effects of negative charge and narrow pore size of *np-ANF*

The pore diameter in the range of single nanometers has two essential benefits for battery membranes related to dendrite suppression and ion transport^46^. First, lithium dendrites have a diameter of around 300 nm in growth point (Fig. 1E, F). Providing sufficient mechanical properties^27,48^, membranes with a single nanometer pore size can suppress the growth dendrites more efficiently than those with wider channels. Notably, the Young’s modulus of *np-ANF* -based membranes no matter in dry (*E* = 9.2 ± 0.5 GPa) or electrolyte wet state (*E* = 8.1 ± 0.4 GPa) are higher than most of previously reported membranes (Supplementary Figs. 8, 9 and Supplementary Table 2). Compared with *Celgard*^*TM*^ 2400 in longitudinal directions with *E* = 0.17 ± 0.02 GPa in dry state and *E* = 0.15 ± 0.02 GPa in electrolyte wet state, it corresponds to *ca* 55x improvement in the Young’s modulus (Fig.1G, Supplementary Fig. 10, and Supplementary Table 1). Even after a long electrochemical looping process, *np-ANF* can still maintain its initial Young’s modulus, while *Celgard*^*TM*^ *2400* reduced significantly and became unstable, which may be caused by dendrite growth on the membrane surface (Supplementary Fig. 10 and Supplementary Table 1). The nano-indentation test further confirms it due to the higher depth of *Celgard*^*TM*^ *2400* membrane compared to the *np-ANF* when the same pressure is applied (Supplementary Fig. 11).

Most importantly, at a high-temperature environment of 80 °C, *np-ANF* still processes a high Young’s modulus of 3.7 GPa, which is much better than the other similar types of separators (Supplementary Fig. 10 and Supplementary Table 1), ensuring the stability of the batteries during charge–discharge operations. The tensile strength and Young’s modulus of *Celgard*^*TM*^ *2400* whether measured in the transverse or longitudinal direction, drop markedly as temperature increase reducing by 70% for Young’s modulus with 0.05 ± 0.003 GPa in a longitudinal direction and 0.4*10^−3^ ± 0.02*10^−3^ GPa in transverse direction at 80 °C. Obviously, it is difficult for *Celgard*^*TM*^ *2400* separator to maintain its original mechanical performance in a high-temperature environment and ensuring the stable operation of the Li–S batteries.

The design criteria regarding Young’s modulus that is easier to measure than many other mechanical characteristics can be derived using the mathematical derivation relating the shear modulus, *G*_polymer_, of the polymeric electrolyte and its Young’s modulus, *E*_polymer_, following the standard Monroe and Newman model^27^. Derivations included in Supplementary Information show that the original expression for the mechanical properties of ion-conducting membranes *G*_polyme*r*_/*G*_Li_ = 1.65 can be converted into *E*_polymer_/*E*_Li_ = 1.53 (Supplementary Information). The advantage of the dendrite suppression relationship based on Young’s moduli is that it is more practical because the values of Young’s moduli are easier to measure and are available for larger number of composite and other materials. Note that this conversion does not make any additional assumptions besides those in the Monroe and Newman model or currently used in the field (Supplementary Information).

Secondly, the negative surface potential of *np-ANF*^39,40,49^ and the small diameter of the pores make the double electric layers extending from the pore walls overlap incompletely compensating the surface charge^50,51^. Being immersed in electrolytes typical for lithium batteries, such membranes are to reject negatively charged chains of LPS due to the repulsion from the channel walls. The nanoscale confinement enables, therefore, a high degree of selectivity for the transport of ions through them re-creating the functional analogs of ion channels known from biology^52,53^.

**Fig. 2:**
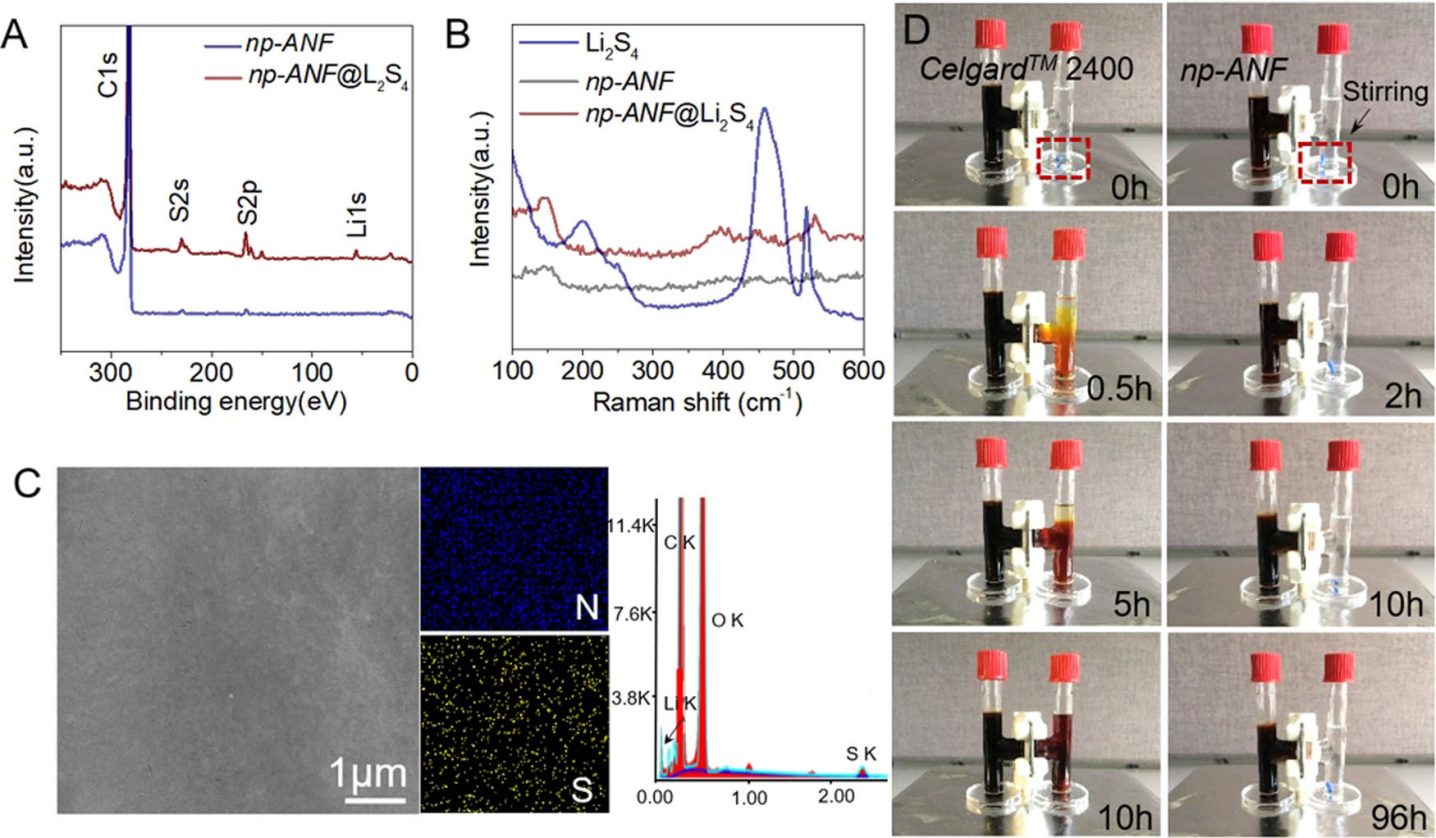
Polysulfide-blocking capability of the *np-ANF* separator. XPS survey (**A**); Raman scattering spectra (**B**); SEM image, EDAX spectra, and the corresponding N and S element mapping images (**C**) for the *np-ANF* before and after adsorption test Li_2_S_4_ solution followed by rinsing with DOL/DME solution and drying in a glovebox. **D** Diffusion of LPS in H-type cell through *Celgard*^*TM*^ 2400 and *np-ANF* membrane.

An additional source of charge on the nanofibers can be the adsorption of chemical species from the electrolyte. We found that LPS can spontaneously bind to *np-ANF* creating the layer of strongly bound negative charge on them, which rejects transport of LPS but facilitates the transportation of lithium ions. *np-ANF* membrane was immersed into 0.5 M Li_2_S_4_ in DOL/DME for 10 h at room temperature (25 °C). After repeated five times wash in copious amount of DOL/DME solvent, a large amount of sulfur was still detected on the surface of the membrane by the XPS analysis (Fig. 2A and Supplementary Figs. 14, 15), indicating the strong adsorption of polysulfides on *np-ANF*. This LPS adsorption on the *np-ANF* membranes was confirmed by EDAX and the Raman scattering (Fig. 2B, C). The two characteristic Raman scattering peaks of LPS observed in freely dissolved states at 450 and 520 cm^−1^ are broadened and appear at 400 and 440 cm^−1^ when adsorbed on *np-ANF*. Broadening is expected for adsorbed LPS due to multiplicity of the conformational states of LPS on *np-ANF* composite.

*np-ANF* composites with negative charges on cylindrical pores^39,49^ are expected to be permeable for lithium ions but reject LPS (Fig. 2D)^54^ due to their negative charge, large size, and cooperative binding to the channel walls. Permeation behavior of LPS through the *np-ANF* membrane was investigated in an H-cell (Fig. 2D) filled with 1,3-dioxolane (DOL) and dimethoxyethane (DME) in 1/1 v/v ratio that is commonly used in the Li–S batteries^55^. DOL/DME solvent without LPS was placed in the right chamber while solvent with 0.5 M Li_2_S_4_ was in the left chamber. Driven by the concentration gradient, LPS easily diffused across the *Celgard*^*TM*^ 2400 and the right chamber turned from colorless to dark brown within 10 h. In contrast, the diffusion of LPS was blocked by *np-ANF* membrane (Fig. 2D) and the right chamber stayed colorless after 96 h even under a strong stirring.

**Fig. 3:**
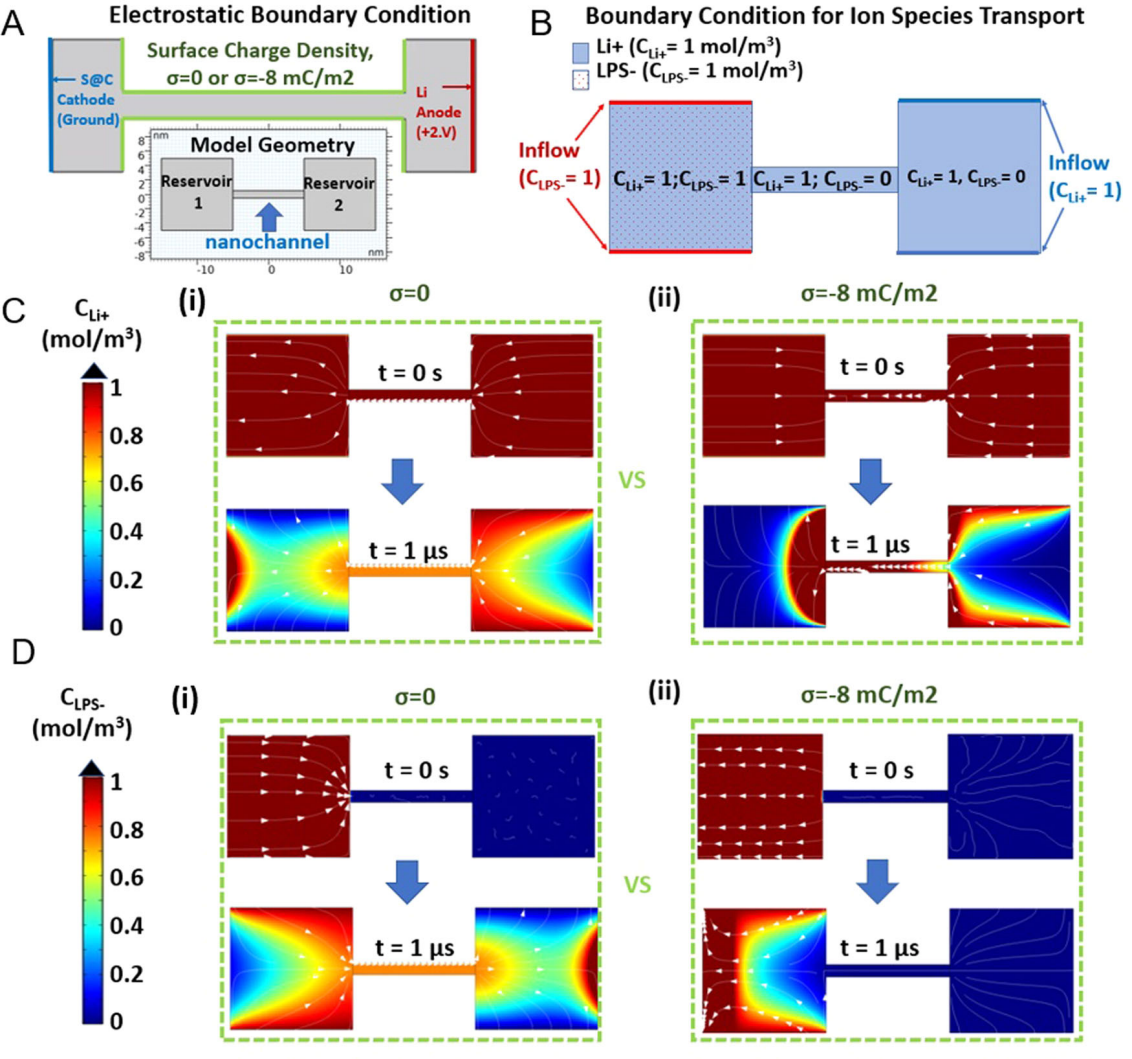
Computational analysis on transport of charged ion species through a single nanochannel. **A** Electrostatic boundary condition including potential set for cathode (ground at S@C, conventional sulfur in carbon) and anode (2 V at Li, Lithium metal), and surface charged density (σ) on ANF wall. **B** Boundary condition for ion transport module for LPS anion and Li cation. The concentration map and streamline change (from *t* = 0 to *t* = 1 μs) of Li cation (**C**) and LPS anion (**D**) with (i) and without (ii) surface charge density on boundary of *np-ANF* separator.

We further investigated LPS blocking mechanism with numerical simulation of ion transport by finite element computations. The structure of the nanochannel of ANF separator was modeled as a single 10 nm long channel with 1 nm diameter connecting two reservoirs (inset, Fig. 3A). The diameter of the model channel has been determined by experimental pore size measured by the BJH analysis (Supplementary Fig. 6) and is similar to the diameter of pores in protein-based ion-channels in the cell membranes. Note that the actual channel is longer than 10 nm and the pore structure is more complex, which represents that our model can even underestimate the selectivity of the transport. Both the reservoirs and nanochannel are represented by dielectric blocks with the electrolyte. The limitation caused by the size of reservoirs of model is compensated by inflow boundary setting of anion and cation (Fig. 3B). The anode and cathode with potential difference of 2 V had been set at the two ends of reservoirs for common electrostatic boundary condition of the models while surface charged density of −8 mC/m^2^ has been selectively applied to the boundary of *np-ANF* separator for comparison. At initial, reservoir on cathode side (reservoir 1, Fig. 3B) is only filled with LPS anions (concentration of LPS-, *c*_LPS_ = 1 M), whereas the whole system including nanochannel is uniformly filled with lithium cation (concentration of Li+, *c*_Li+_ = 1 M). Two different electrostatic conditions, with and without surface charge density on the surface of *np-ANF* membrane, were applied to the model and time-dependent studies were performed by solving the coupled Poisson, Nernst–Planck, and Navier–Stokes equations (Supplementary Information). As expected, both LPS anion and Li cation can freely pass through the nanochannel that does not have a surface charge (Fig. 3C, D, I and Supplementary Movies 1, 2). When the model includes, however, the negative surface charge on the channel walls, the transport of LPS including some of the smallest LPS species, such as including Li_2_S_4_, is blocked (Fig. 3D, ii and Supplementary Movie 4). Li^+^ cations still pass though unimpeded (Fig. 3C, ii and Supplementary Movie 3). Note that until 200 ns, some adsorption of Li cation layer on the wall of *np-ANF* has been observed although it does not block the passage of ion transport. Ion transportation simulation model through large pores (>20 nm has also been modeled) to evaluate the performance of *Celgard*^TM^ for LPS leakage (Supplementary Fig. 12). Thus, the computational model demonstrates that *np-ANF* can selectively block LPS transport solely due to electrostatic repulsion, even excluding the size selectivity effect, which simplifies the engineering of the membranes for a variety of technologies.

### Ion-selective *np-ANF* membranes enable Li–S batteries with high sulfur loading

**Fig. 4:**
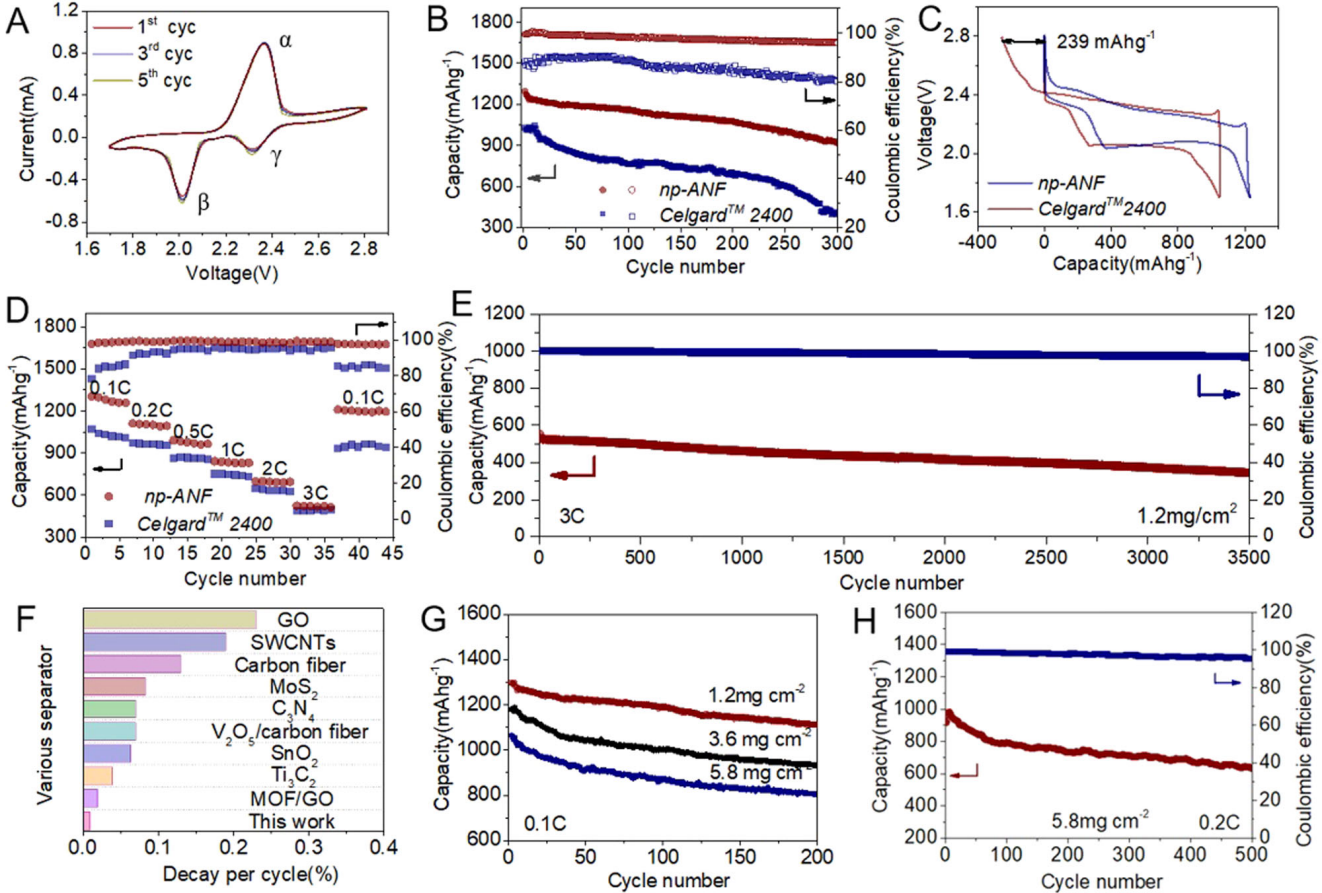
Electrochemical performance of the Li–S batteries with *np-ANF* and *Celgard*^*TM*^ 2400 membrane. **A** CV profiles with *np-ANF* at a scan rate of 0.1 mV s^−1^; **B** Cycling performance comparison of Li–S batteries with *np-ANF* and *Celgard*^*TM*^ 2400 membrane at a rate of 0.1C; **C** Galvanostatic charge–discharge profiles of *np-ANF* and *Celgard*^*TM*^ 2400 membrane at a rate of 0.1C; **D** Rate performance of Li–S batteries ranged 0.1C to 3C with *np-ANF* and its comparison of *Celgard*^*TM*^ 2400; **E** Cycling performance Li–S batteries with *np-ANF* membrane at a rate of 3C over a period of 3500 cycles. **F** The decay per cycle of Li–S batteries with various membrane Table S5); **G** Cycling performance of Li–S batteries at 0.1C at various sulfur loading. **H** Cycling performance Li–S batteries with *np-ANF* membrane at a rate of 0.2C after 500 cycles at sulfur loading of 5.8 mg cm^−2^.

In the cyclic voltammograms (CV) of Li–S battery cell with *np-ANF* membranes, two distinctive reduction peaks and a strong oxidation peak appear at 2.36, 2.02, and 2.31 V, respectively (Fig. 4A). For simplicity of notations, the anodic peak and the two cathodic peaks will be denoted here as peaks α, β, and γ, respectively. The peak *γ* at 2.36 V is attributed to the reduction of S_8_ to intermediate LPS (Li_2_S*x*, 4 ≤ *x* ≤ 8), while the second reduction peak *β* at 2.02 V is ascribed to the further reduction of intermediate LPS to insoluble Li_2_S and Li_2_S_2_. The strong oxidation peak α centered at 2.31 V corresponds to the delithiation of Li_2_S/Li_2_S_2_ into Li_2_S*x* (4 ≤ *x* ≤ 8) and eventually to S_8_^5^. In the subsequent cycles, the intensities of reduction and oxidation peaks remain almost unchanged.

Using the classical Randles−Sevick equation^3,56^, one can understand further Li^+^ ion diffusion properties, acquiring CV curves under different scanning rates ranging from 0.1 to 0.5 mV s^−1^. The diffusion coefficients were determined to be *D*_Li+_(α_1_) = 9.263•10^−8^ cm^2^s^−1^, *D*_Li+_(β_1_) = 5.310•10^−8^ cm^2^s^−1^, and *D*_Li+_ (γ_1_) =0.421•10^−8^ cm^2^s^−1^ for *np-ANF*, which almost equal to the diffusion coefficients for the *Celgard*^*TM*^ 2400 of *D*_Li+_(α _2_) = 9.693•10^−8^ cm^2^s^−1^, *D*_Li+_ (β_2_) = 5.192•10^−8^ cm^2^ s^−1^, and *D*_Li+_ (γ_2_) = 0.567•10^−8^ cm^2^ s^−1^, respectively (Supplementary Fig. 15 and Supplementary Table 3). The lithium-ion transference number (*t*_Li+_*)* of *np-ANF* separator (0.63) is almost similar to that pure *Celgard*^*TM*^ *2400* (0.68) (Supplementary Fig. 16 and Supplementary Table 3). These data indicate that *np-ANF* does not affect the diffusion properties of Li^+^. Linear sweep voltammetry (LSV) indicates the high electrochemical stability of *np-ANF* in Li–S batteries (Supplementary Fig. 17), suggesting that *np-ANF* separator are suitable for Li–S batteries.

Diffusion of LPS through membranes is detrimental effects for Coulombic efficiency of Li–S batteries even for low charge/discharge rate of 0.1C. Coulombic efficiency is less than 70−90% for common Li–S batteries. Coulombic efficiency of the cells being cycled at 0.1C for 300 cycles as 98 and 75% after for *np-ANF* and *Celgard*^*TM*^ 2400, respectively (Fig. 4B). Besides Coulombic efficiency, the discharge capacity also increased accordingly because the *np-ANF* membranes hindered the chemical reactions between metallic lithium and high-order LPS, reducing the loss of active material loss. For example, the initial discharge capacity of 1268 mAh g^−1^ obtained on the cell with an *np-ANF* was higher than that for *Celgard*^*TM*^ 2400 with 1029 mAh g^−1^ (Fig. 4C). Furthermore, the capacity decay rate was also reduced from 0.20% of *Celgard*^*TM*^ 2400 membrane to 0.092% per cycle. Comparing to other membranes designed for Li–S batteries, *np-ANF* membranes have an advantage because they improve the cycle life for long term at different charge/discharge rate (Supplementary Table 5). When needed, the cycle life, as well as the Coulombic efficiency, could be further enhanced by increasing the thickness of *np-ANF*. However, it will increase the diffusion path length and internal resistance (Supplementary Fig. 18)^57,58^ with concomitant reduction of capacity. One can expect that various energy consumption patterns will impose different preferences to Li–S battery operations. The ability of *np-ANF* to address different requirements will be essential for the future multiparameter performance optimization of charge storage devices.

Electrochemical impedance spectroscopy (EIS) of the Li–S cells were interpreted using equivalent circuits in Supplementary Fig. 19. Batteries with *np-ANF* membranes showed a more considerable resistance compared to *Celgard*^*TM*^ 2400 before cycling (Supplementary Fig. 19A), which is expected considering the reduced pore size in these ion-conductive membranes. However, after 100 cycles (Supplementary Fig. 19B), the *R*_ct_ value of the cell with *np-ANF* significantly decreases, which may be resulted from infiltrating of the electrolyte and the chemical activation of the active materials^59^. For comparison, the battery with *Celgard*^*TM*^ 2400 after 100 cycles shows two semicircles (Supplementary Fig. 19B). The semicircle in high frequency (*R*_sf_//CPE_sf_) is related to the formation of an insulating layer of solid Li_2_S_2_/Li_2_S on lithium anode^60^ and the second semicircle in low frequency (*R*_ct_//CPE_ct_) is assigned to the charge transfer resistance. On the contrary, the batteries with *np-ANF* membranes possess only one semicircle in the high-frequency region (red curve in Supplementary Fig. 19 and Supplementary Table. 4). Furthermore, the corresponding *R*_ct_ value is lower for *np-ANF* than for *Celgard*^*TM*^ 2400, which indicates faster reaction kinetics due to the formation of the SEI film on the electrode’s surface making the Li ions transport easier^61,62^.

Although the transport of LPS is blocked, Li^+^ ion transport through *np-ANF* is not and, in fact, remains fast. Li^+^ ion conductivity is as high as 0.24 mS/cm due to the low internal resistance, favorable electrolyte wettability, and high electrolyte uptake (Supplementary Figs. 4, 5), which translates into excellent rate performance of Li–S cell when ion-selective when *np-ANF* is used as ion conductor (Fig. 4D and Supplementary Fig. 20). With the current densities varied between 0.1 and 3.0C, discharge capacities of 1268, 1092, 969, 889, 703, and 521 mAh g^−1^ were observed. When the charge/discharge rate was reduced back to 0.1C, a capacity of 1224 mAh g^−1^ close to the original one was recovered for the cell with *np-ANF* whereas 931 mAh g^−1^ was observed for the cell with *Celgard*^*TM*^ 2400 membrane. The batteries utilizing np*-ANF* membranes display a cycle life over 3500 cycles at 3C (Fig. 4E) with a capacity decay as low as 0.01% per cycle (Fig. 4F). Compared to many other ion-conductive membranes used for Li–S batteries, including carbon interlayers that serve mainly as physical barriers, *np-ANF* is more efficient in the long-term inhibition of LPS diffusion (Supplementary Fig. 21 and Supplementary Table 5).

Moreover, to evaluate the feasibility of the *np-ANF* separator in electric vehicles and similarly demanding applications, the sulfur loading was increased to 3.6 and 5.8 mg cm^−2^. The batteries were still able to deliver a high initial capacities of 1142 mAh g^−1^ (3.6 mg cm^−2^) and 1018 mA h g^−1^ (5.8 mg cm^−2^) (Fig. 4G) with the corresponding areal capacities of 4.1 and 5.9 mAh cm^−2^. Based on the charge/ discharge curves (Supplementary Fig. 22A), no polarization increase upon higher sulfur loading was observed. We also tested the cells with high sulfur loading for various current rates from 0.1C to 3.0C (Supplementary Fig. 22B). A high discharge capacity of 558 mAh g^−1^ at 3.0C was attained. Similarly to the case of 1.2 mg cm^−2^ sulfur loading, switching the charge–discharge current back recovers the capacity of the battery to 940 mAh g^−1^ even when the sulfur loading as high as 5.8 mg cm^−2^ due to effective mitigation of LPS transfer from cathode to anode. Long-term cycling for 500 cycles (initial capacity 945 mAh g^−1^, 5.5 mAh cm^−2^ at 0.2C) is also possible for high-sulfur cathodes (Fig. 4H); their capacity is competitive or better than most Li–S cells with sulfur loading ≥3.0 mg cm^−2^ (Supplementary Table 6).

### *np-ANF* membranes engender high-temperature tolerance of Li–S batteries

**Fig. 5:**
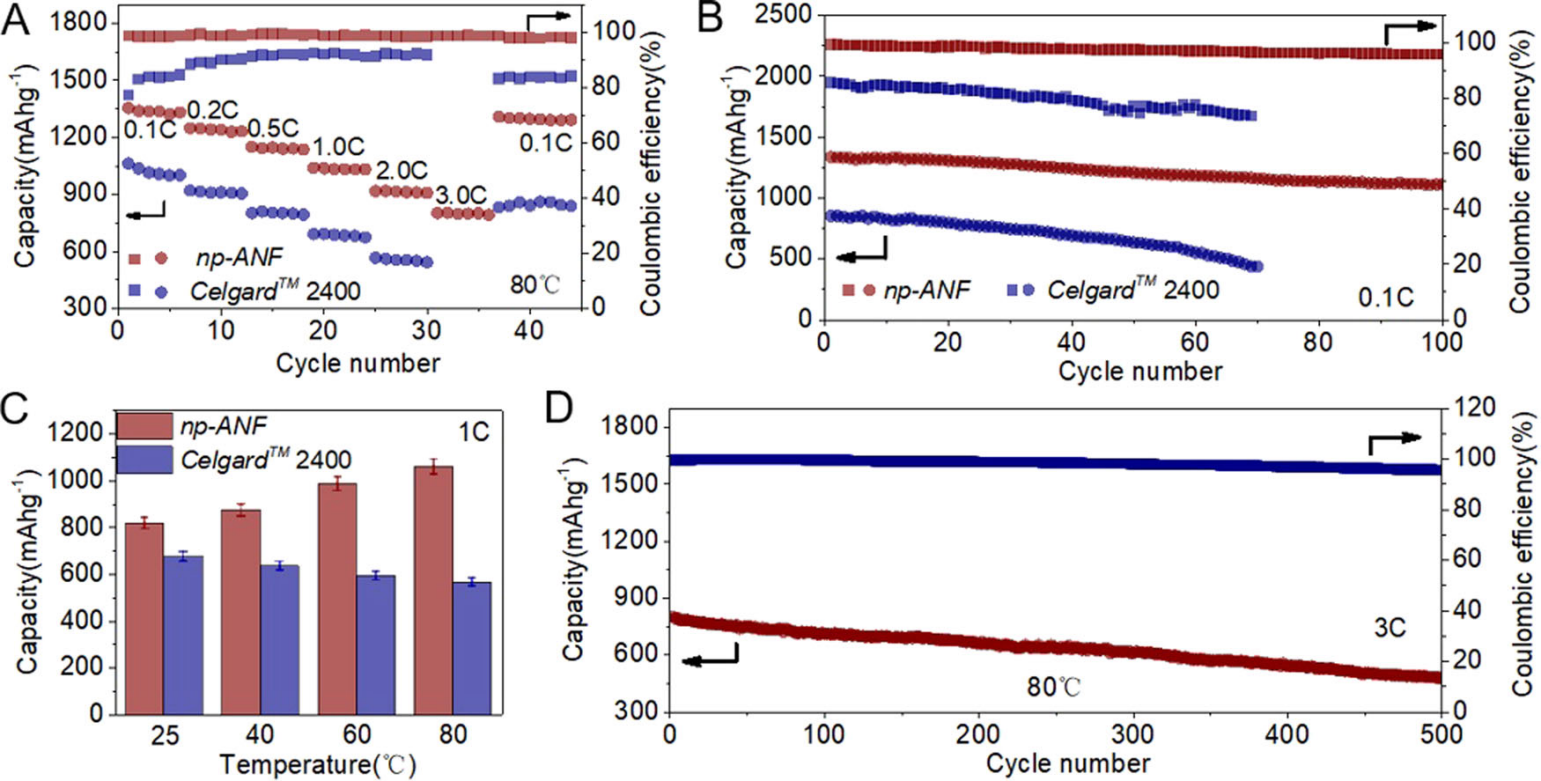
Electrochemical performance of the Li–S batteries with *np-ANF* and *Celgard*^*TM*^ 2400 membrane at elevated temperature of 80 °C. **A** Rate performance of Li–S batteries ranged 0.1C to 3C with *np-ANF* and its comparison of *Celgard*^*TM*^ 2400. **B** Cycling performance of Li–S batteries at 0.1C at 80 °C. **C** The capacity comparison of *np-ANF* and *Celgard*^*TM*^ 2400 membrane at different temperatures. **D** Cycling performance Li–S batteries with *np-ANF* membrane at a rate of 3C after 500 cycles at 80 °C.

The tolerance of a Li–S batteries to elevated temperatures is an important parameter determining their safety, as the increased operational temperature is accompanied with higher charge-discharge rates.  Futhermore, increased operational temperature  can also aggravate the dissolution of polysulfides, accelerating the consumption of active materials and the attenuation of capacity. Thus, the batteries with *np-ANF* membranes were evaluated at 80 °C. The *np-ANF* cells show an *increase* in capacity to 1346 mAh g^−1^ at 0.1C (Fig. 5A) compared to room temperature cycles (1268 mA hg^−1^)^26,63^. When the current density increased stepwise, the discharge capacities of the *np-ANF* were 1249, 1140, 1032, 916, and 801 mA hg^−1^ at 0.2C, 0.5C, 1C, 2C, and 3C, respectively, with a capacity retention of around 60%, exhibiting a much better high-temperature rate performance than that of cell with *Celgard*^*TM*^ *2400* membranes. When the charge/discharge rate was reduced back to 0.1C, a discharge capacity of 1302 mAh g^−1^ was observed and almost kept steady for the subsequent 100 charge–discharge cycles whereas the capacity decreased rapidly and fell to below 300 mAh g^−1^ after only 60 cycles for the cell with *Celgard*^*TM*^ *2400* membrane (Fig. 5B). The capacities of cell employing *np-ANF* increase due to the increased diffusion coefficient of Li^+^ in polysulfides (Fig. 5C), whereas an opposite trend was observed for *Celgard*^*TM*^ *2400* membrane due to the rapid self-discharge caused by crossover of LPS. The battery with *np-ANF* also display long cycle life with a low-capacity decay (0.081% per cycle) over 500 cycles at 3C and the Coulombic efficiency remain 95−99% (Fig. 5D) at temperatures as high as 80 ^o^C, which exceeds the state-of-the-art of Li–S batteries (Supplementary Table 7).

### *np-ANF* membranes suppress Li dendrites

**Fig. 6:**
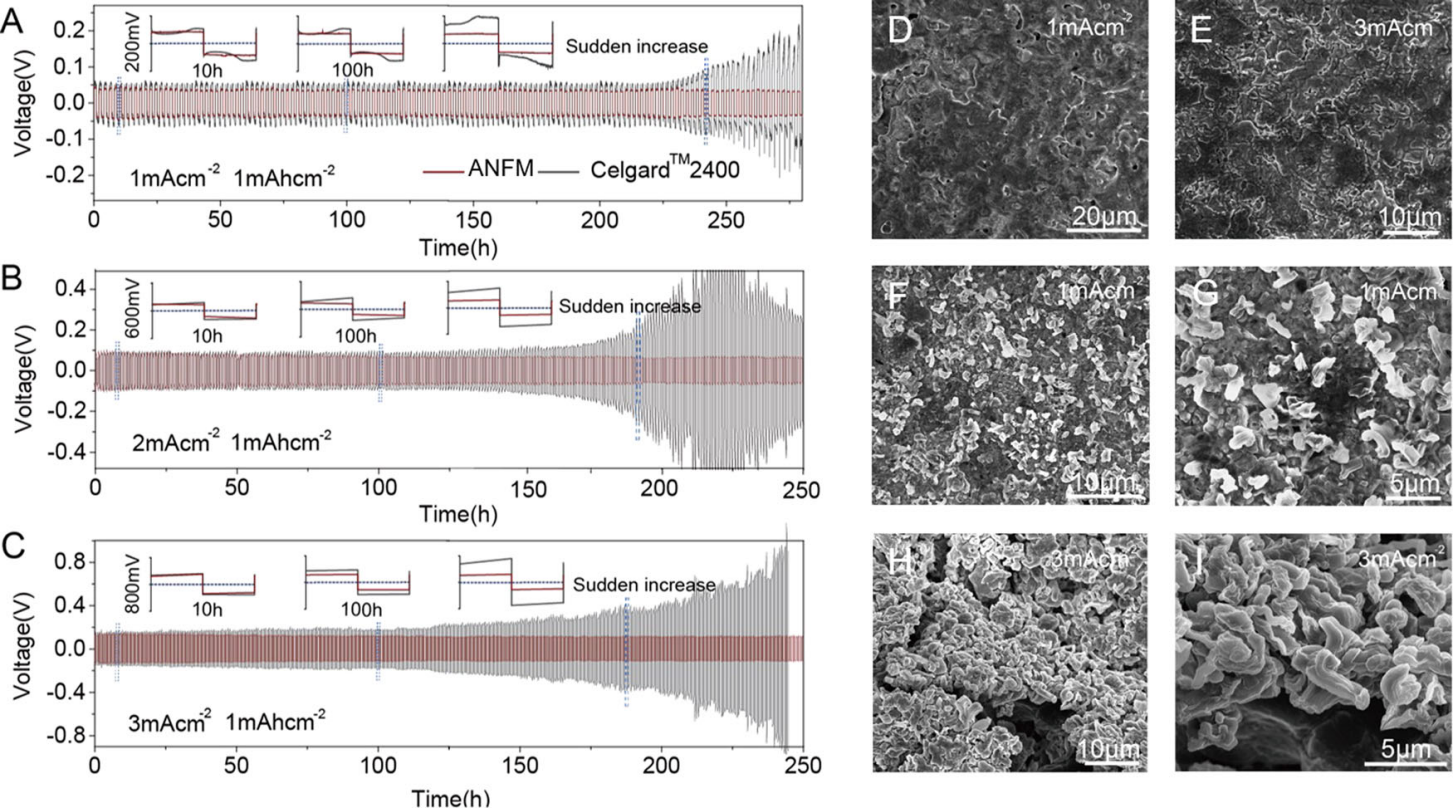
Comparison of the cycling stability of the *np-ANF* and the *Celgard*^*TM*^ 2400 membranes at a current density of **A** 1 mA cm^−2^, **B** 2 mA cm^−2^, and **C** 3 mA cm^−2^ with a stripping/plating capacity of 1 mAh cm^−^^2^. SEM images of the lithium electrode with *np-ANF* membrane after 250 h cycles of stripping/plating in 1 mol/L LiCF_3_SO_3_ DOL: DME v/v = 1/1) at a current density **D** 1  mA cm^−2^ and **E** 3 mA cm^−2^. SEM images of the lithium electrode with *Celgard*^*TM*^ 2400 membrane after 250 h cycles of stripping/plating in 1 mol/L LiCF_3_SO_3_ DOL: DME v/v = 1/1 at a current density (**F**), magnified image (**G**) 1 mA cm^−2^ and (**H**), and magnified image (**I**) 3 mA cm^−2^.

All Li metal anodes suffer from dendrite growth upon charging. To demonstrate dendrite suppression by *np-ANF* membrane, cyclic charge/discharge in a symmetrical Li/membrane/Li with 1 mol/L LiCF_3_SO_3_ DOL: DME (v/v = 1/1) cell were evaluated over 250 h (Fig. 6A). The cell with *np-ANF* exhibited negligible loss or fluctuation of voltage. In contrast, the voltage of cells from *Celgard*^*TM*^ 2400 increased over cycles, by almost 100% after 250 h due to dendrite-induced soft short circuit. To study the evolution of the voltage profiles in detail, the 10 h 100 h and sudden increase cycling of the cells with *np-ANF* and *Celgard*^*TM*^ 2400 membrane were further enlarged as the insets in Fig. 6A. Flat voltage profiles at both the charging and discharging states can be retained throughout the whole cycle without obvious increases in hysteresis for cells with *np-ANF* membranes, whereas batteries with *Celgard*^*TM*^ 2400 showed fluctuating voltage profiles with consistently higher overpotential at both the initial and final stages of each stripping/plating promoting the growth of dendrites. Greatly improved cycling stability was also observed with *np-ANF* as we increased the current density to 2 mA cm^−2^ and 3 mA cm^−2^ process (Fig. 6B, C), whereas the cells with *Celgard*^*TM*^ 2400 exhibited a gradual increase of the hysteresis. Although high rate charge/discharge cycling stimulates the dendrite growth, the surface image of lithium electrode remained consistently flat even at a high current density of 3 mA cm^−2^ after 250 h cycling (Fig. 6E). Lithium cells with *Celgard*^*TM*^ 2400 displayed rough surface and massive formation of dendrites after 250 h cycling under the same conditions or even at a lower current density 1 mA cm^−2^ (Fig. 6F, G). At a high current density of 2 and 3 mA cm^−2^, extensive growth of dendrites was observed on lithium electrode surface (Fig. 6H, I and Supplementary Fig. 23). Such drastically different results further demonstrate the advantages of *np-ANF* membranes.

### Multifactorial performance for Li–S batteries with *np-ANF* membranes

**Fig. 7:**
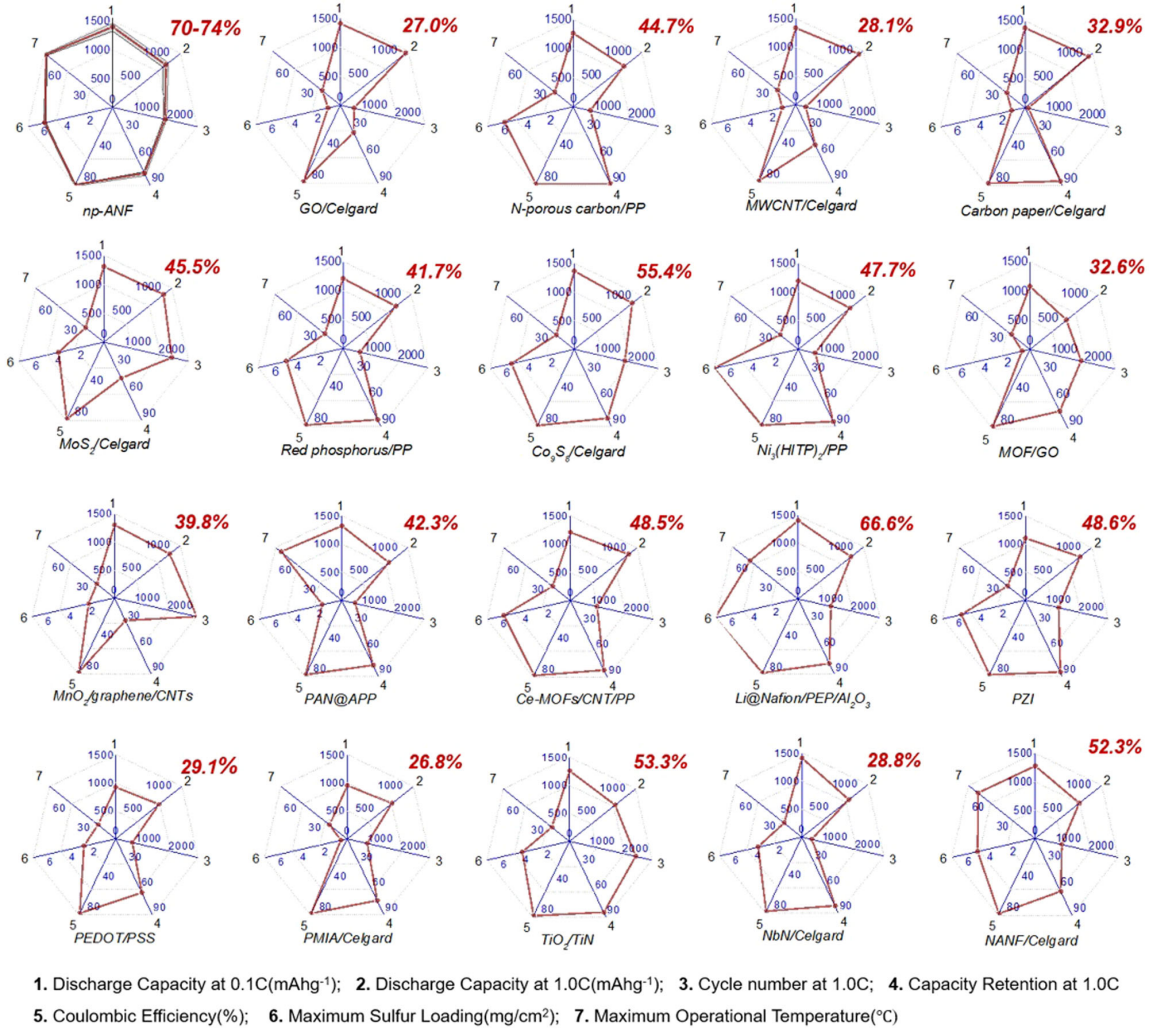
Multiparameter comparison of various for Li–S batteries based on glyph plots and cumulative capability criterion (CCC). The value of CCC is given in the top red corner of each glyph plot. The detailed data set for the plots are provided in Supplementary Information in Table S8. The gray area in *np-ANF* in the top left corner is the variability range for our measurements. For many performance parameters, the error value was not reported.

As an example of a complex task to engineer a battery satisfying several often contrarian parameters such as high discharge rate and long cycle life, we carried out multiparameter performance assessment of Li–S cells with *np-ANF* separators using glyph plots (Fig. 7). The following parameters were chosen to evaluate the state-of-the-art Li–S batteries due to their significance to multiple technologies: initial discharge capacity at 0.1C (mAh g^−1^); initial discharge capacity at 1.0C (mAh g^−1^); cycle life at 1.0C; capacity retention at 1.0C (%); Coulombic efficiency (%); maximum sulfur loading (mg/cm^2^); maximum operational temperature (°C). Since battery capabilities increase with the increase of these metrics, the total area encircled by the glyph plot can be used as a cumulative capability criterion (CCC, %) to compare and optimize the batteries. CCC is enumerated as a percentage of the area encircled by the glyph plot from the maximum possible for the capability-relevant range of properties (Fig. 7). When feasible, it can also include uncertainty intervals based on the reported measurement errors. Interdependent variations of different parameters obtained for different versions of similar materials or different operation conditions may also be included. Here we analyzed the data for Li–S batteries with comparable data sets. The assessment shows that cells with *np-ANF* membranes have across-the-board performance exceeding the current state-of-the-art in Li–S batteries. When needed, a longer list of the parameters can be selected, albeit methodology will remain the same. For the broader use of CCC analysis, the incorporation of the variability range as we included for our data is recommended. It can be particularly important for the large parameter sets when several materials can be similar in the CCC analysis so that several engineering options can be determined.

## Discussion

Biomimetic *np-ANF* membranes integrating ion-channel-line nanoscale pores and  cartilage-like architecture can effectively block LPS transport while remaining transparent to Li^+^ ions. In addition to LPS screening based on ion size enabled by the 1 nm pores in *np-ANF*, surface charge density on the walls of the pores is enhanced by adsorption of LPS that repell similar ions. The combination of a highly interconnected network of percolated nanofibers and intrinsic materials properties of aramid simultaneously enable effective suppression of dendrites and long-term operation of the batteries at elevated temperatures. The multifactorial engineering of *np-ANF* membranes encompassing narrow pore size, negative charges, high mechanical and thermal properties collectively lead to the promising electrochemical performance in Li–S batteries exceeding the current state of the art comparing several singular parameters and across-the-board performance. The simplicity of *ANF* fabrication and availability of parent material Kevlar as a recyclable polymer enables their broad utilization in different energy technologies.

## Methods

### Preparation of the ANFs and ion-selective membranes from them

To prepare the ANF dispersion, the fabrication method is reported previously^49^. 1 g of bulk Kevlar 69 (from Thread Exchange) and 1.5 g KOH were added into 500 mL of dimethyl sulfoxide (DMSO) which was magnetically stirred for two weeks at room temperature forming a dark red solution of ANF. For LBL assembly of nanoporous ANF-based membranes abbreviated here as *np-ANF*, microscope glass slides were cleaned in piranha solution (3:1 H_2_SO_4_/H_2_O_2_) for 24 h, followed by thorough rinsing with deionized water before use. After that, setting a clean piece of the glass slide on the disk of the spin coater, 2 mL of ANF (2 wt%) solution was dropped and spin-coated at 1500 rpm for 30 s. After spin coating, the membrane was immediately put into DI water to remove the DMSO and then dried at 70 °C in oven 10 min. Then the glass slide was dipped into 0.1 wt% poly(diallyldimethylammonium chloride) (PDDA) solution to change the charge for 1 min and rinsed with water for 2 min before air drying. This sequence of steps was repeated three times and the last layer is ANF. It should be noted that the thickness of membrane can be ranged by increase or decrease the membrane’s layer. The membrane is peeled from the substrate by immersing in 0.1% (hydrofluoric acid) HF solution. The remaining chemicals were purchased from Sigma Aldrich without further purification. ANF dispersion with similar composition and characteristics of the nanofibers can also be made from previously used Kevlar^TM^ fabrics, fibers, and yarns. The process of their recycling into ANF will include the process of dissolution in DMSO in presence of KOH identical to that described above.

### Structural characterization

The morphology of the Li dendrite, *Celgard*^*TM*^ 2400, and *np-ANF* membrane were inspected with a scanning electron microscope (SEM, FEI Nova Nanolab dual-beam FIB). The *np-ANF* before and after Li_2_S_4_ adsorption was measured by Raman spectra (LabRAM HR 800 spectrometer at 785 nm) and X-ray photoelectron spectra (XPS, Thermo Fisher Scientific, USA. The data were obtained in an XPS system, via monochromatic Al Kα radiation with a pass energy of 50 eV.) Note: *The np-ANF* membrane was immersed into 0.5 M Li_2_S_4_ in DOL/DME for 10 h at room temperature (25 °C) followed by repeated pure DOL/DME solution washing to remove any residual LPS and then dried in glovebox.

Quantachrome Autosorb 6B system was used to characterize the pore width of the *np-ANF* membrane using nitrogen sorption under 77.4 K. The pore size distributions of the *np-ANF* membrane were calculated by BJH methods. The contact angle was measured by Cam‐plus Micrometer with the sessile drop technique.

Electrolyte uptake tests were conducted by measuring the weights of the sample before (*W*_dry_) and after (*W*_wet_) soaking in liquid electrolyte (1 M LiTFSI, DOL /DME (v/v = 1/1) for 2 h and calculated according to equation:

$${{{{{\rm{Electrolyte}}}}}}\,{{{{{\rm{uptake}}}}}}=\frac{{W}_{{{{{{\rm{wet}}}}}}}-{W}_{{{{{{\rm{dry}}}}}}}}{{W}_{{{{{{\rm{dry}}}}}}}}\times 100 \%$$

For dimensional and volumetric change analysis, sample thickness was measured using a caliper, and the area was measured using digital imaging before and after electrolyte uptake.

The mechanical tests of the membranes were performed on a TA XT Plus Texture Analyzer (Stable Micro Systems Ltd.) instrument at room temperature and high temperature of 80 °C, with the test of longitudinal direction carried out at a rate of 2 mm min^−1^. The film was cut into rectangular strips of 20 mm × 5 mm, and twenty samples were tested in each case. For wet conditions, the sample was soaked into electrolyte solution (1 M LiTFSI, DOL /DME (v/v = 1/1) for 2 h before testing. Dry conditions did not soak into the electrolyte solution. The mechanical performance of the separator after the electrochemical testing also have been tested. The separator was taken from cells that have been evaluated in a symmetrical Li/membrane/Li system over 250 h at a current density of 1 mA cm^−2^ with the electrolyte of 1 mol/L LiCF_3_SO_3_ DOL: DME (v/v = 1/1), The nano-indentation tests of membranes were performed on a Bruker Hysitron TI980 instrument at room temperature, and five points were tested in each sample.

The thermal stability of membrane was analyzed by TA Instruments Discovery thermogravimetric analyzer (TGA) with a temperature ramp to 700 at 10 °C min^−1^ in nitrogen at a flow rate of 30 mL min^−1^ and DSC with a temperature ramp to 500 at 10 °C min^−1^ in nitrogen at a flow rate of 30 mL min^−1^.

### Visualization of ion-selectivity

The electrochemical cell test that cab visualize LPS blocking was carried out in an H-type glass cell to examine the properties of the membrane. *Celgard*^*TM*^ 2400 and *np-ANF* membranes were set in the middle of glass cell. The left chamber was filled with 0.5 M Li_2_S_4_ solution with DOL /DME (v/v = 1:1) as the solvent, while the right chamber was only filled with DOL/DME solvent (v/v = 1:1). The Li_2_S_4_ was prepared by a synproportionation reaction^64^ between Li_2_S and S with a mole ratio of 1:2.

### Computation for the transport of charged ion species

To elucidate the mechanism of selective ion transport through the *np-ANF* membrane, we have simplified the system as a single nanopore channel of the np-ANF membrane connecting two reservoirs. The 2D nanochannel model was implemented in the commercial finite element solver, COMSOL Multiphysics.

#### Model geometry

The system consists of electrolyte reservoirs connecting through the 10 nm long nanochannel. The diameter of the channel was set to 1 nm based on the experimental pore size determined by the BJH analysis (Supplementary Figs. 5, 6). Both the reservoirs and nanochannel are represented by dielectric blocks with the electrolyte.

#### Governing equations

The dynamics of our system can be solved by the coupled Poisson, Nernst–Planck, and Navier–Stokes equations, a well-known set of partial differential equations that represents the electrostatic field, the ionic flux, and the fluid flow, respectively^65–68^.

#### Electrostatic field

The electrostatic potential of the system has evaluated using Poisson’s equation, solved using the electrostatic interface in COMSOL. For the potential, Poisson’s equation states:

$$\nabla \cdot ({\varepsilon }_{0}{\varepsilon }_{{r}}\nabla \phi )=-\rho ,$$

where *ϕ* is the electric potential, *ε*_*0*_ the vacuum permittivity (8.85419 × 10^−12^ F · m^−1^), *ε*_*r*_ relative permittivity of the material, and *ρ* total charge density which was derived from zeta potential measurements.

#### Ionic flux

The total ionic flux *J*_*i*_ of each ion, *i*, is governed by the Nernst−Planck equation, which can be expressed as the sum of diffusive, electrophoretic, and convective fluxes:

$${J}_{i}=-[{D}_{i}\nabla {c}_{i}+{z}_{i}{\mu }_{\iota }{c}_{i}\nabla \phi -{{{{{\boldsymbol{u}}}}}}{c}_{i}],$$

with *D*_*i*_ being the ion diffusion coefficient, ${c}_{i}$ the ion concentration, ${z}_{i}$ the ion charge number, ${\mu }_{i}$ the electrophoretic mobility of ion *i*, and ***u*** the fluid velocity.

And assuming no homogeneous reaction in the electrolyte, at steady state, the governing equations for the species become:

$$\frac{{{{{{\rm{\delta }}}}}}{c}_{i}\,}{{{{{{\rm{\delta }}}}}}{t}}=-\nabla \cdot {J}_i=0,$$

#### Fluid flow

The fluid flow model is set as an incompressible flow regulated by the Navier–Stokes equations:

$${\pounds} \frac{{{{{{\rm{\delta }}}}}}{{{{{\boldsymbol{u}}}}}}\,}{{{{{{\rm{\delta }}}}}}{t}}+({{{{{\boldsymbol{u}}}}}}\cdot \nabla )\,(\pounds {{{{{\boldsymbol{u}}}}}})=\nabla \cdot [-{p{{{{{\bf{I}}}}}}}+{{{{{{\bf{K}}}}}}}]+{{{{{{\bf{F}}}}}}},$$

with the hydrodynamic stress tensor, **K** = *η* [∇***u*** + (∇***u***)^T^] and the continuity equations, £ ∇·***u*** = 0, where £ is the fluid density, *η* the dynamic viscosity, *p* the pressure, **F** is the volume force vector.

#### Multiphysics coupling

Other than potential coupling from electrostatic to the transport of diluted species interface, space charge density coupling has been added by defining dependence of the space charge density (SI unit: C/m^3^), *ρ*, on the ion concentrations:

$$\rho =F{\sum }_{i}{z}_{i}{c}_{i}$$

with *F* Faraday’s constant (96485.33 C · mol^−1^), and *c*_*i*_ the ion concentration and ${z}_{i}$ ion charge number of ion *i*.

### Electrochemical performance tests

Standard 2032-coin cells (MTI) were used to evaluate the electrochemical performance of the *np-ANF* membrane-incorporated Li–S batteries. The C/S cathode materials were fabricated with a typical melt-diffusion strategy. The nanocarbon was firstly mixed with sulfur powder with a mass ratio of 3:6 by milling. The mixture was subsequently placed in a sealed flask at 155 °C for 10.0 h to incorporate S into the carbonaceous matrix. The cathode slurry was then prepared by mixing 90 wt% sulfur/carbon composite and 10% poly(vinylidene fluoride) binder in N-methylpyrrolidone (NMP) solvent dispersant. The cathode was constructed by coating the slurry on aluminum foil and drying at 60 °C at vacuum oven for 24.0 h. The sulfur loading was 1.2 mg cm^−2^ for the regular tests and around 3.6 and 5.8 mg cm^−2^ for the high sulfur loading test. Cathode samples were punched into 16 mm diameter circles. The metallic lithium foil (thickness 0.5 mm, diameter 16 mm) purchased from MTI Corporation was directly used as the anode. Then 1 M LiTFSI solution in DOL/DME (v/v = 1/1) was used for the cells and the electrolyte/sulfur ratio was controlled at 10:1 (μL mg^−1^). The separator used in the cell were routine *Celgard*^*TM*^ *2400* (thickness 25 μm) or *np-ANF* (thickness 5.8 μm). Separator samples were punched into 19 mm diameter circles. The coin cells were tested in galvanostatic mode at various currents within a voltage range of 1.7–2.8 V using LAND-CT2001A battery-testing instrument. Both CV and EIS were performed on an Autolab (Autolab Potentiastat and Solartron 1260 frequency response analyzer). The EIS carried out in the range from 100 kHz to 0.05 Hz with potential amplitude of 20 mV. The resulting Nyquist plots were fitted to an equivalent circuit where ionic conductivity was then calculated from the equation: σ = *L*/*R*_b_*A*, where *L* is the thickness of the film, *R*_b_ is the bulk resistance, and A is the contact area of film. The CV was scanned at a rate range of 0.10–0.50 mV s^−1^. LSV was conducted using an Autolab. The separator was placed in between a stainless-steel electrode and a Li foil working electrode (both 16 mm in diameter) with 1 M LiTFSI in DOL/DME (v/v = 1/1) as the electrolyte. The cell was then held at 0 V for 5 min and then ramped from 0 to 4 V at a scan rate of 0.5 mV s^−1^. The capacities were calculated based on the mass of sulfur in the cathode.

The lithium-ion diffusion coefficient *D*_Li+_ (cm^2^ s^−1^) was evaluated by cyclic voltammetry and calculated according to the Randles–Sevick equation:^3,56,69^

1 $${I}_{{{\mbox{p}}}}=2.69\times {10}^{5}{n}^{1.5}A{D}_{{{{{{\rm{Li}}}}}}^+}^{0.5}C{v}^{0.5}$$

in which *I*_p_ (A) is the peak current, *n* represents the number of electrons of in the reaction (for Li–S batteries, *n* = 2), *A* (cm^2^) indicates the electrode area (1.54 cm^2^ here), *C*_Li+_ (mol/mL) means the lithium-ion concentration(0.1 mol/L) in the electrolyte, and *v* is the scanning rate (Vs^−1^).

The Li^+^ transference number (*t*_Li*+*_*)* for different separators was evaluated by a potentiostatic polarization method of a constant potential at 10 mV was applied for 10,000 s to record the current at initial and steady-state. Each membrane was separately sandwiched between two lithium metal electrodes in a coin-type cell (CR2023) and saturated with 1 M LiTFSI in DOL/DME (v/v = 1:1) electrolyte. The Li^+^ transference number was calculated from the ratio of steady-state current to initial state current according to the following equation:

2 $${t}_{{{{{{\rm{Li+}}}}}}}={I}_{{{{{{\rm{s}}}}}}}/{I}_{0}$$

where *t*_Li*+*_ is transference number, while *I*_s_ and *I*_0_ represent the current at the steady state and initial state, respectively.

### The relationship between Young’s Modulus and dendrite suppression

The model developed by Monroe and Newman shows that dendrites become unstable when the interfacial stability parameter Δ*µ* changes from positive to negative^27^. For the interface between lithium and polyethylene oxide (PEO), this transition occurs when

3 $${{G}}_{{{{{{{\rm{PEO}}}}}}}}/{{G}}_{{{{{{{\rm{Li}}}}}}}}=1.65,$$

where *G*_PEO_ and *G*_Li_ are shear moduli of the polymeric electrolyte and lithium metal, respectively.

While the shear modulus *G* is related to Young’s modulus, *E*, as

4 $${G}=0.5\,{E}/(1+{\nu })$$

where *ν* is the Poisson’s ratio of the material. In case of metallic Li and PEO, *ν*_Li_ = ~0.4 and *ν*_PEO_ = 0.33, respectively, leading to *G*_Li_ *=* 0.5 *E*_Li_/(1 *+* 0.4) and *G*_PEO_ *=* 0.5*E*_PEO_/(1 + 0.33). Then, the threshold condition *G*_PEO_/*G*_Li_ = 1.65 for Li/PEO interphase transforms into 1.4*E*_PEO_/1.33*E*_Li_ = 1.65 or

5 $${{E}}_{{{{{{{\rm{PEO}}}}}}}}/{{E}}_{{{{{{{\rm{Li}}}}}}}}=1.53$$

Note that the threshold condition in Eq. 3 is dependent on Poisson’s ratios of materials on both sides of the interface, as can be seen by examining Figs. 3, 4, and 6 in the original paper by Monroe and Newman^27^. However, the current battery literature conceptualizes Eq. 3 as a condition for dendrite suppression not just for PEO but for any material disregarding the dependence of the *Δµ* threshold and respect *G*_polymer_/*G*_Li_ on Poisson’s ratio of the polymer. Examples of such approaches in the current electrochemical literature are multiple^70–75^.

While being a deviation from the original expression for displacement functions (Eq. 16 from 2005 Monroe-Newman study)^27^, such simplification is driven by materials properties used in practice is justified because the Poisson’s ratio of solid semi-crystalline and amorphous polymers vary in a narrow range of values^76–78^. The same logical path can be taken considering Eq. 5 leading to a modification of the original expression Eq. 3 as *E*_polymer_/*E*_Li_ = 1.53. Note that this expression is consistent with the Monroe-Newman framework of continuum mechanics and the assumption of small deformations and Hookean-elastic regime as well as subsequent elaborations on the theory of dendrite growth^27,79^. Some of these points were also discussed in our prior publication^39^.

## Supplementary information

Supplementary Information

Description of Additional Supplementary Files

Movie S1 Li

Movie S2 LPS

Movie S3 Li

Movie S4 LPS

## Data Availability

The experiment data that support the findings of this study are available from the corresponding authors upon reasonable requests.
