# Supplementary material for: Multifactorial engineering of biomimetic membranes for batteries with multiple high-performance parameters
Source: Nat Commun. 2022 Jan 12;13:278. doi: 10.1038/s41467-021-27861-w (PMC8755825; doi:10.1038/s41467-021-27861-w)
Supplement: Supplementary file 1 — Supplementary Information [file 41467_2021_27861_MOESM1_ESM.pdf]

Supplementary Information

Multifactorial engineering of biomimetic membranes for batteries with multiple high-performance parameters

Mingqiang Wang[1,2,3,ϕ], Ahmet Emre[2,3,4,5,ϕ], Ji-Young Kim[2,3,4], Yiting Huang[1], Li Liu[1], Volkan Cecen[2,3], Yudong Huang[1], Nicholas A. Kotov[2,3,4,5,*]

[1] School of Chemistry and Chemical Engineering, Harbin Institute of Technology, Harbin 150001, P. R. China;

[2] Department of Chemical Engineering, University of Michigan, Ann Arbor, Michigan 48109, USA;

[3] Biointerfaces Institute, University of Michigan, Ann Arbor, Michigan 48109, USA;

[4] Department of Materials Science and Engineering, University of Michigan, Ann Arbor, Michigan 48109, USA;

[5] Department of Biomedical Engineering, University of Michigan, Ann Arbor, Michigan 48109, USA;

[ϕ] These authors contributed equally: Mingqiang Wang, Ahmet Emre.

*Correspondence: kotov@umich.edu

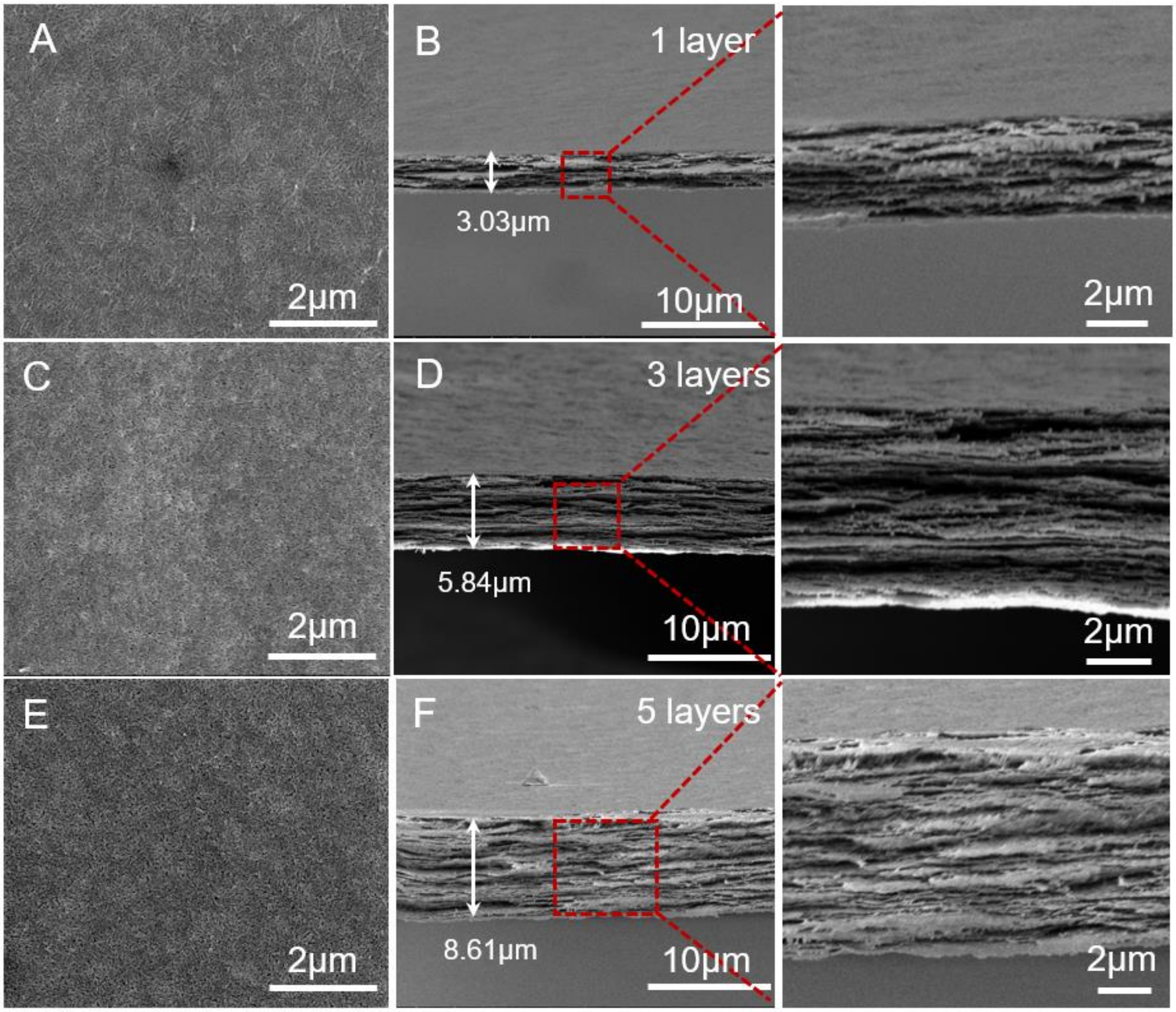

Supplementary Fig 1. (A to F) The SEM images of the surface view and side view of *np-ANF* membrane with different thickness made by sequential deposition of nanofiber strata. (A and B) *np-ANF* membrane with one layer, (C and D), *np-ANF* membrane with three layers, (E and F), *np-ANF* membrane with five layers. SEM images with enlarged sections of the membranes are given in the third column.

While we used LBL assembly to engineer the *np-ANP* composite, the single bilayers (i.e. ANF+PDDA) are thicker than the typical nanometer-scale bilayers from polyelectrolytes observed in LBL-made materials in the past. There is a distinct possibility that ANFs are depositing in the non-linear (exponential) fashion, which can lead to the gradual increase in layer thickness over multiple consecutive cycles due to diffusion-in/diffusion-out mechanism.[1–3]

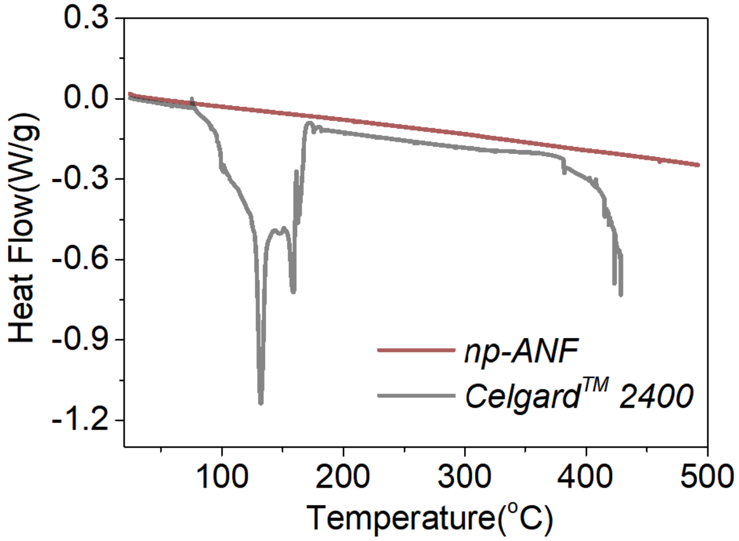

Supplementary Fig 2. DSC curves comparison of *np-ANF* and *Celgard*^{TM} 2400 membrane. The *np-ANF* membrane shows excellent thermal stability with no obvious phase change until 500°C. The superior thermal tolerance could effectively prevent internal short-circuit at elevated temperature. In comparison, *Celgard*^{TM} 2400 decomposes at 300 °C and exhibits an endothermic peak at 122 °C.

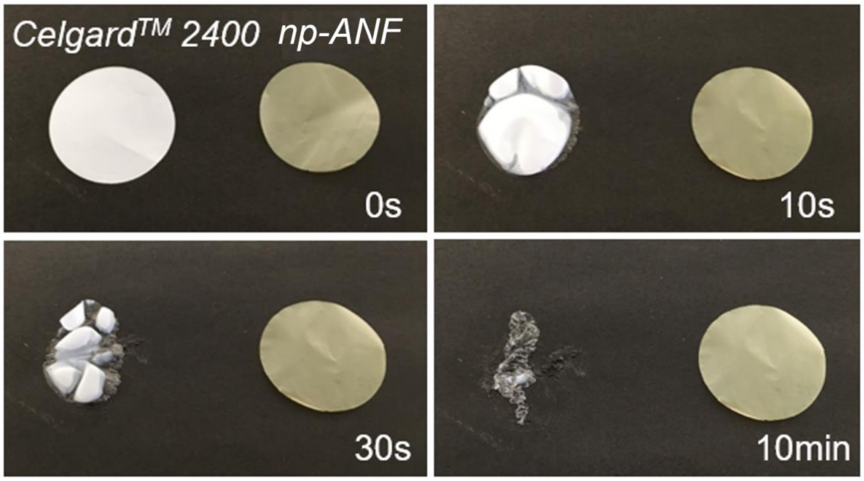

Supplementary Fig 3. Hot solder iron test on *np-ANF* and *Celgard*^{TM} 2400 membrane at 150℃ with increase time. The time-lapse photography demonstrates a clear difference between two membranes of *np-ANF* and *Celgard*^{TM} 2400 on a 150 °C hot plate. The *np-ANF* remained unchanged, showing good thermal stability, while *Celgard*^{TM} 2400 membrane shrank strongly.

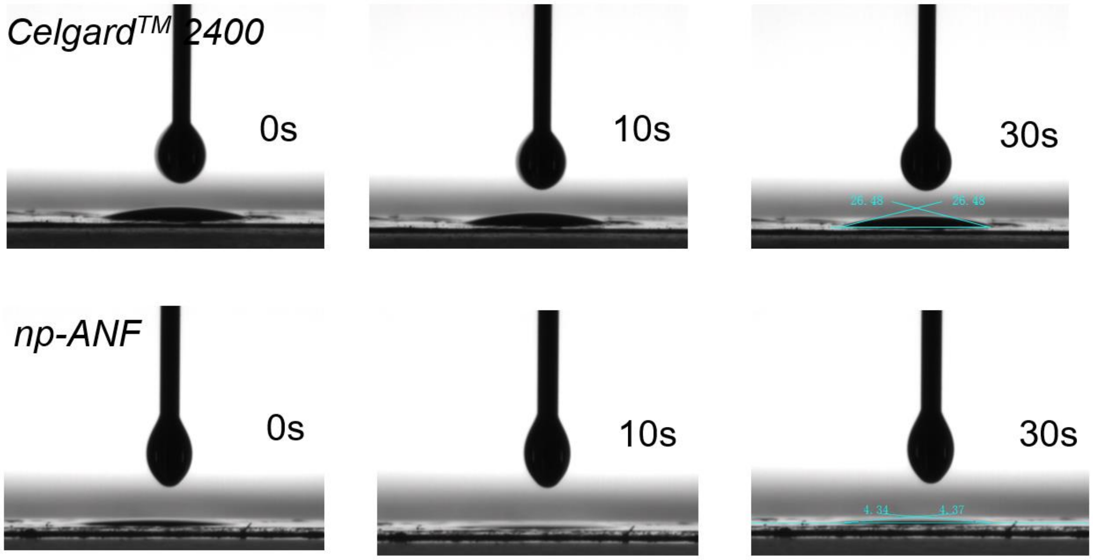

Supplementary Fig 4**.** Photographs of static liquid electrolyte contact angles of different separators at different rest time. 1 M LiTFSI with 2 wt% $LiNO_3$ in DOL/DME (v/v=1:1) was used as a liquid electrolyte.

Besides the lower electrolyte contact angle of np-ANF in comparison to *Celgard™ 2400*, the *np-ANF* also has a higher electrolyte uptake.

The electrolyte uptake in this work was evaluated by the weights of sample before (W_{dry}) and after (W_{wet}) soaking in liquid electrolyte (1 mol/L LiTFSI, DOL /DME (v/v= 1/1) for 2 h and calculated according to equation:

$$E\text{lectrolyte uptake}=\frac{W_{wet}-W_{dry}}{W_{dry}}\times 100\%$$

The electrolyte uptake of the pristine *Celgard™ 2400* and the *np-ANF* separator are 158% and 328%, respectively. The greatly improved electrolyte uptake is due to nanofibers were stacked to form a 3D network porous structure as well as the good affinity to lipid groups in the electrolyte[4].

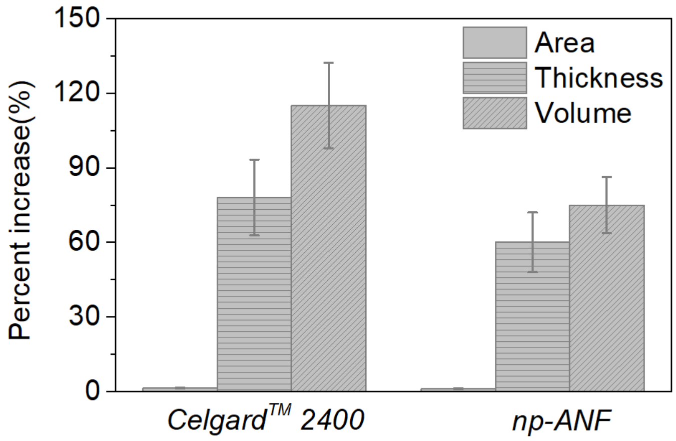

Supplementary Fig 5. Dimensional changes in *Celgard*^{TM} *2400* and *np-ANF* when soaked in electrolyte solution

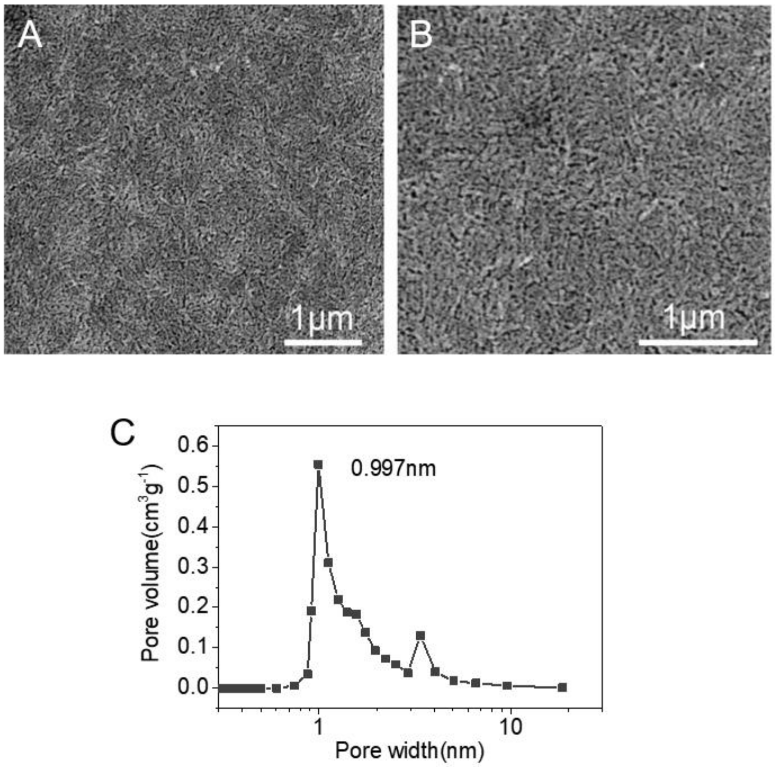

Supplementary Fig 6. (A-B) The magnified SEM images of the surface view *np-ANF* membrane made after three deposition cycles. (C) Pore size width distributions of the *np-ANF* membrane obtained from Barrett-Joyner-Halenda (BJH) analysis.

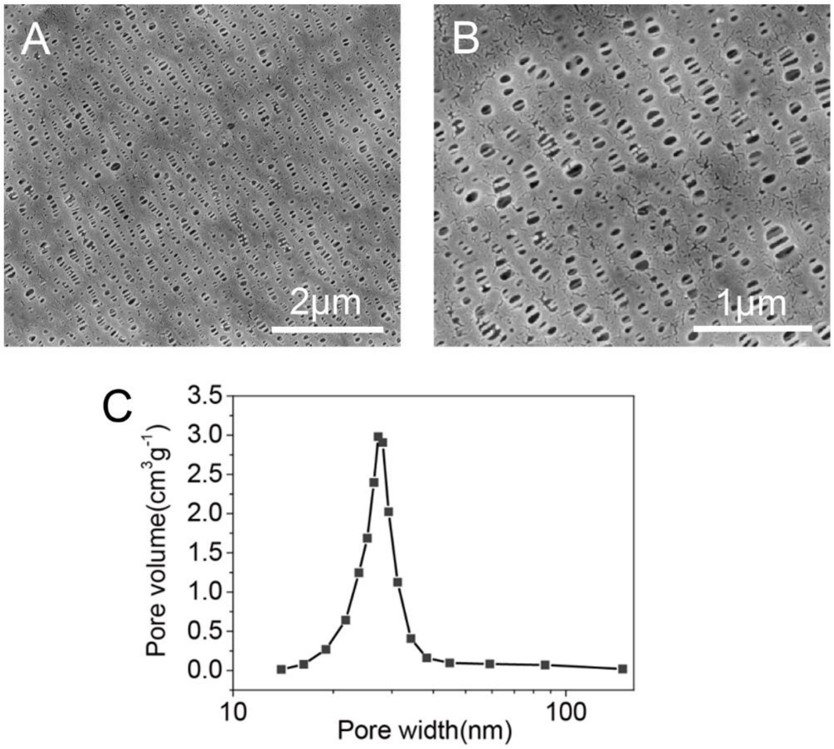

Supplementary Fig 7**.** (A-B) SEM image of surface of *Celgard*^{TM} 2400. (C) The pore size width distributions of the *Celgard*^{TM} 2400 obtained from Barrett-Joyner-Halenda (BJH) analysis.

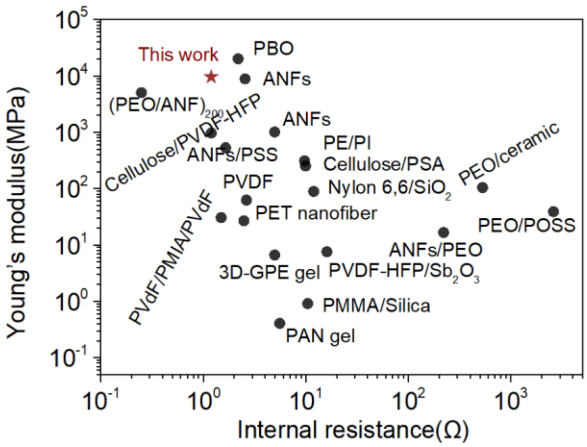

Supplementary Fig 8. Comparative evaluation of Young's modulus and internal resistance normalized to a standard CR2032 coin cell for *np-ANF* and other membranes. The internal resistance of *np-ANF* membrane is obtained by electrochemical impedance spectroscopy (EIS) curve of Li/ *np-ANF* /Li in DOL/DME solution with a standard CR2032 coin as shown in Figure S9. The corresponding references and the list of abbreviations are given in Table S2.

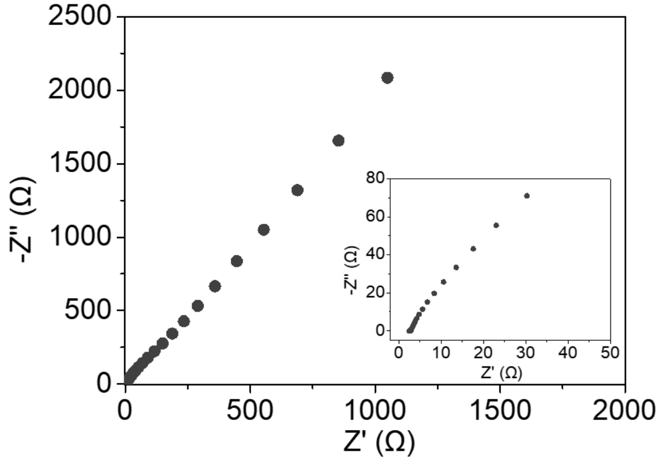

Supplementary Fig 9. The impedance spectra of Li/ *np-ANF* /Li using standard CR2032 coin cell.

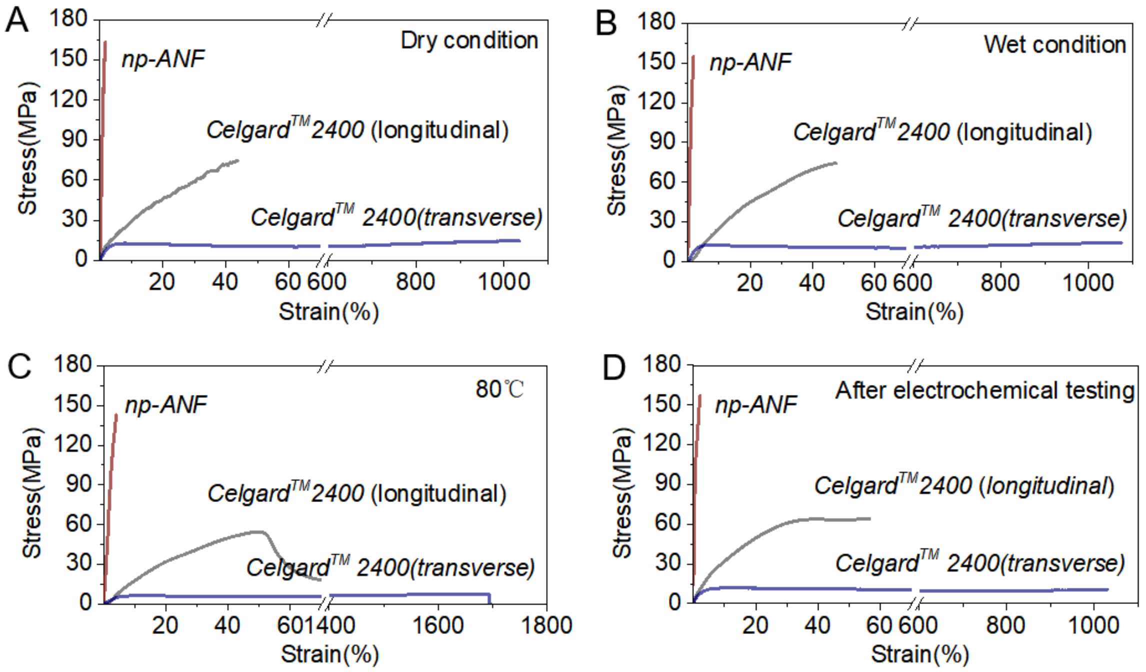

Supplementary Fig 10**.** Stress-strain curves for *np-ANF* and *Celgard*^{TM} 2400 in different state (A) dry (B) wet; (C) 80℃; (D) after electrochemical testing.

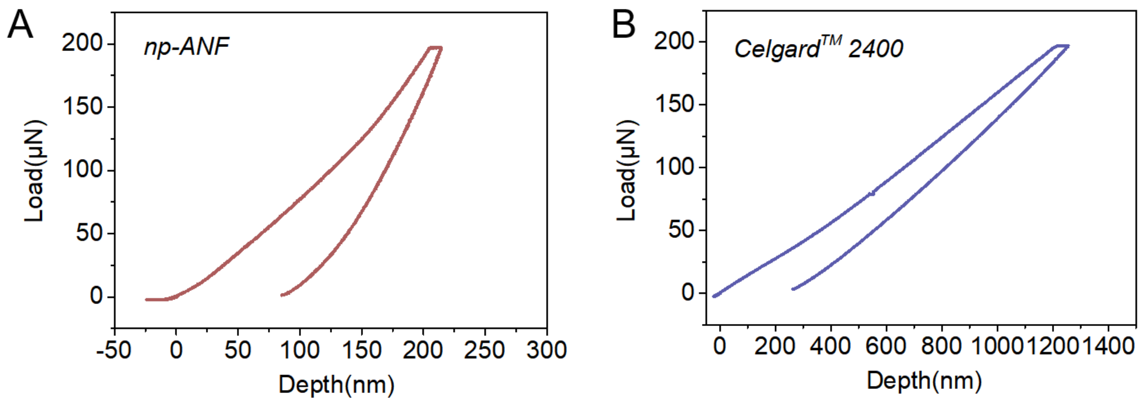

Supplementary Fig 11. Nano-indentation comparison for (A) *np-ANF* and (B) *Celgard*^{TM} 2400

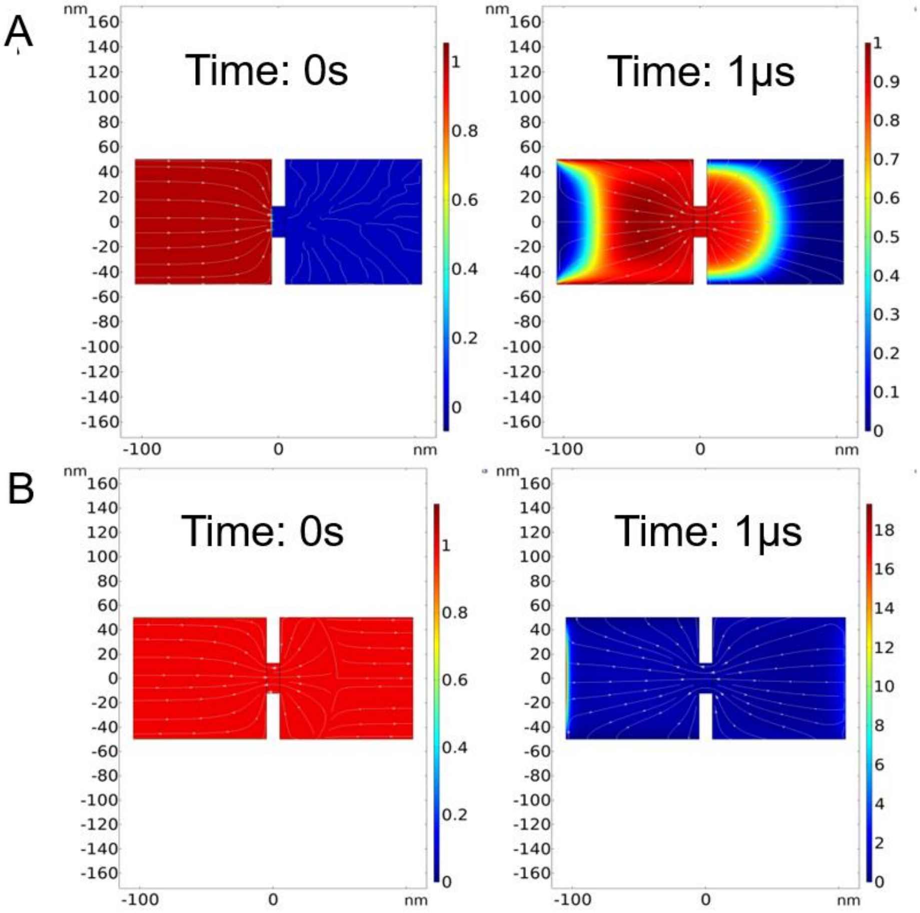

Supplementary Fig 12. Theoretical simulation of ionic flow in *Celgard*^{TM} separator pores by finite element analysis (COMSOL Multyphysics). The concentration map and streamline change of LPS (A) and Li ion (B) from t = 0 to t = 1 μs.

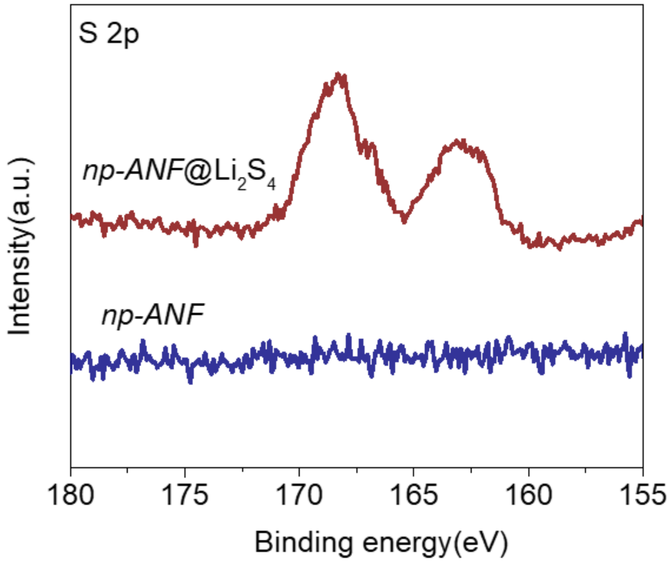

Supplementary Fig 13. the High-resolution XPS spectra of S_{2p} comparison of *np-ANF* before and after adsorption test Li_2S_4 solution followed by rinsing with DOL/DME solution and drying in glovebox

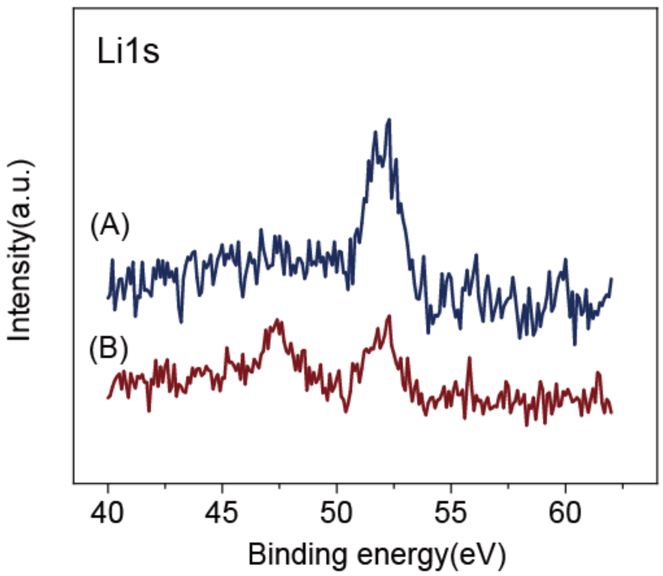

Supplementary Fig 14. High-resolution XPS spectra of retrieved *np-ANF* sample (A) from $LiNO_3$ solution (B) from Li_2S_4 solution.

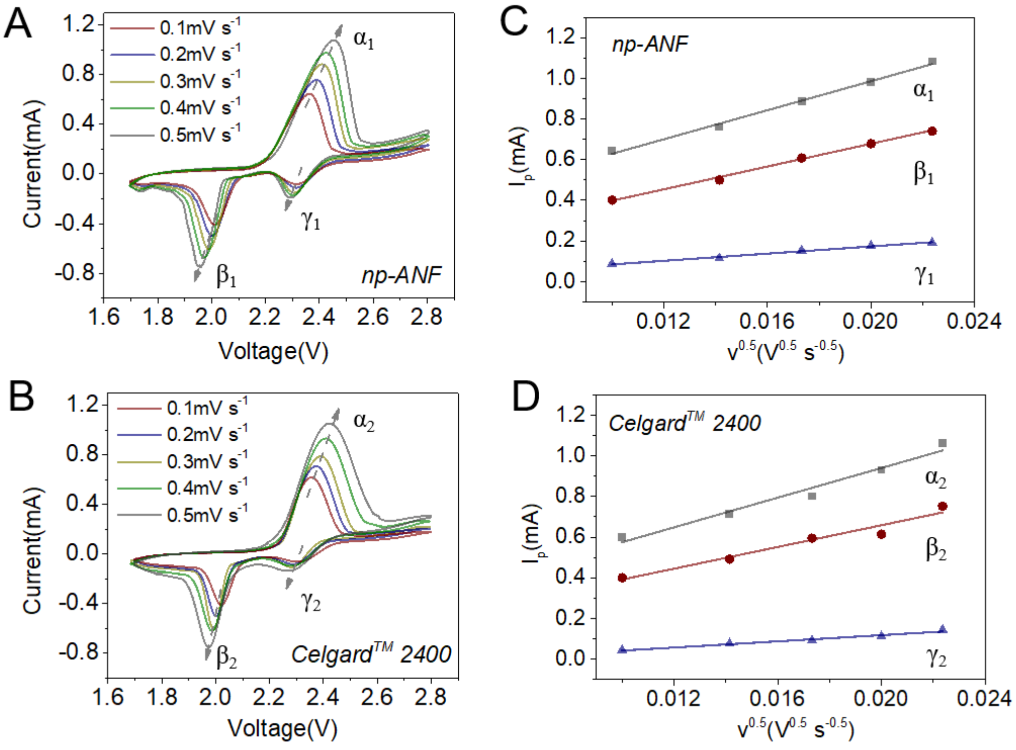

Supplementary Fig 15. Cyclic voltammograms at different voltage scan rates of Li-S cells: (A) with *np-ANF* membrane and (B) with *Celgard*^{TM} 2400 membrane; (C-D) the linear fits of the peak currents for cells with *np-ANF* (C) and *Celgard*^{TM} 2400 (D) membranes.

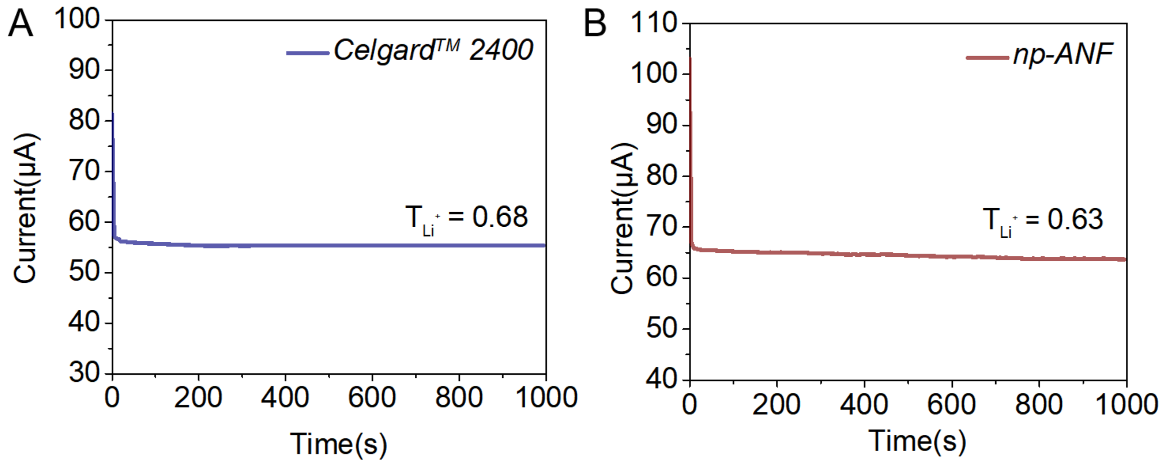

Supplementary Fig 16**.** Lithium ions transference number for (A) *Celgard^{TM}* 2400; (B) *np-ANF*

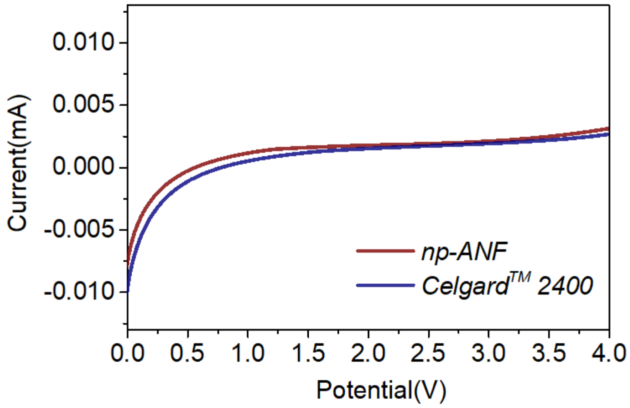

Supplementary Fig 17. Linear sweep voltammetry for *Celgard™ 2400*, and *np-ANF* separators. The cell configuration was a two electrode cell consisting of Li metal working electrode and a stainless-steel counter electrode with a scan rate of 0.1 mV s^{-1}.

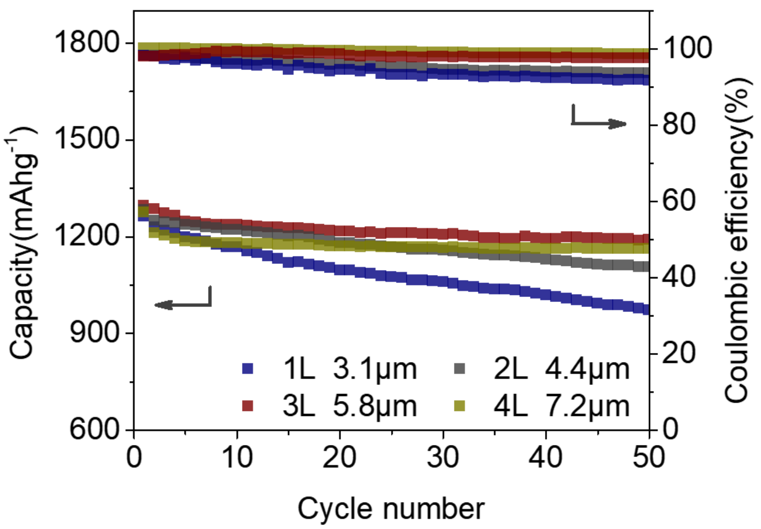

Supplementary Fig 18. Cycling performance of Li-S batteries with *np-ANF* membranes with from one layer to four layers at 0.1C rate.

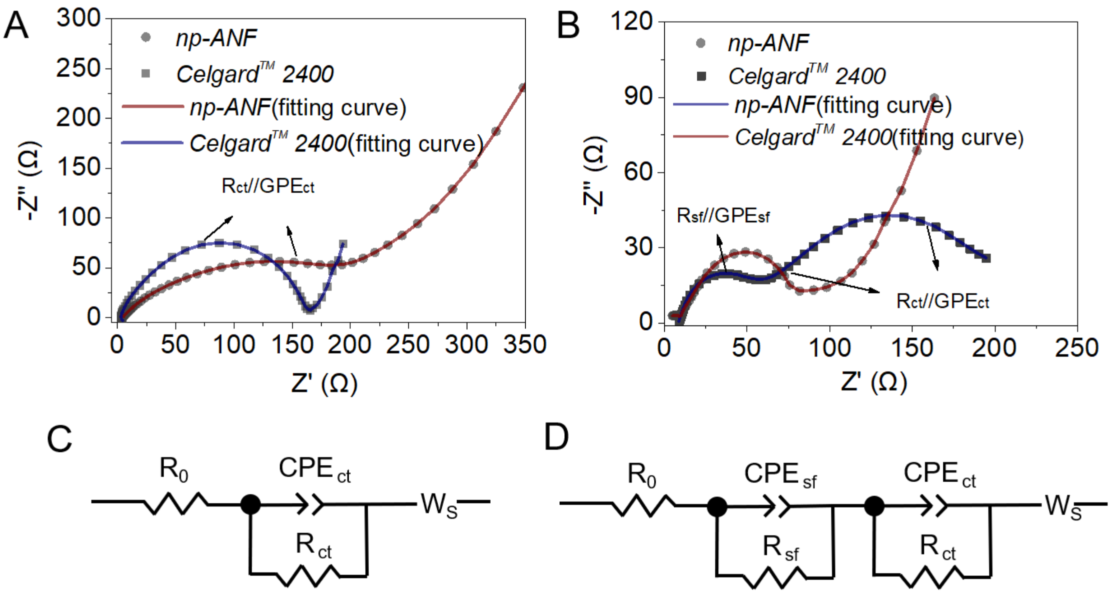

Supplementary Fig 19. Electrochemical impedance spectra of Li–S batteries (**A**) before and (**B**) after cycling using *Celgard*^{TM} 2400 and *np-ANF* membranes, and corresponding equivalent circuits before (**C**) and after (**D**) cycling; notations: R_0 is interphase-contact resistance of the electrolyte and battery; R_{ct} is the charge transfer resistance; R_{sf} is the surface film resistances; W_s is the Warburg impedance, CPE is a constant-phase element (CPE) attributed describing the double layer capacitance [5,6]

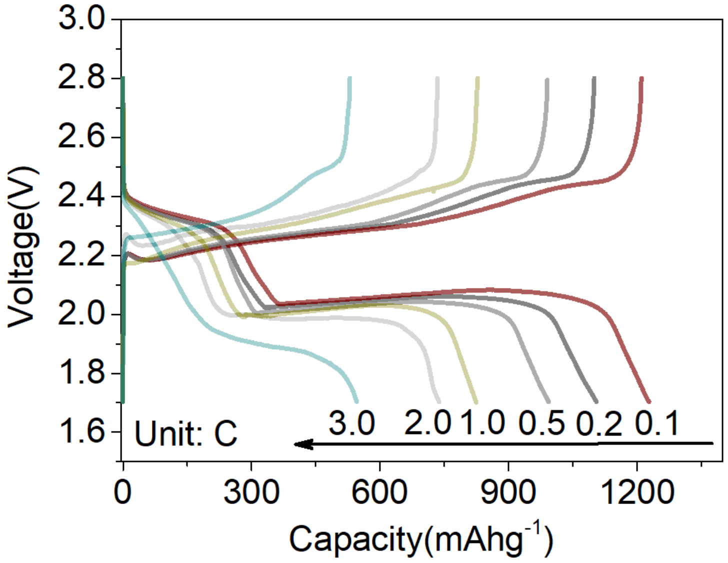

Supplementary Fig 20. Rate performance of Li-S cell with *np-ANF* membrane at a sulfur loading of 1.2mg/cm^{-2}.

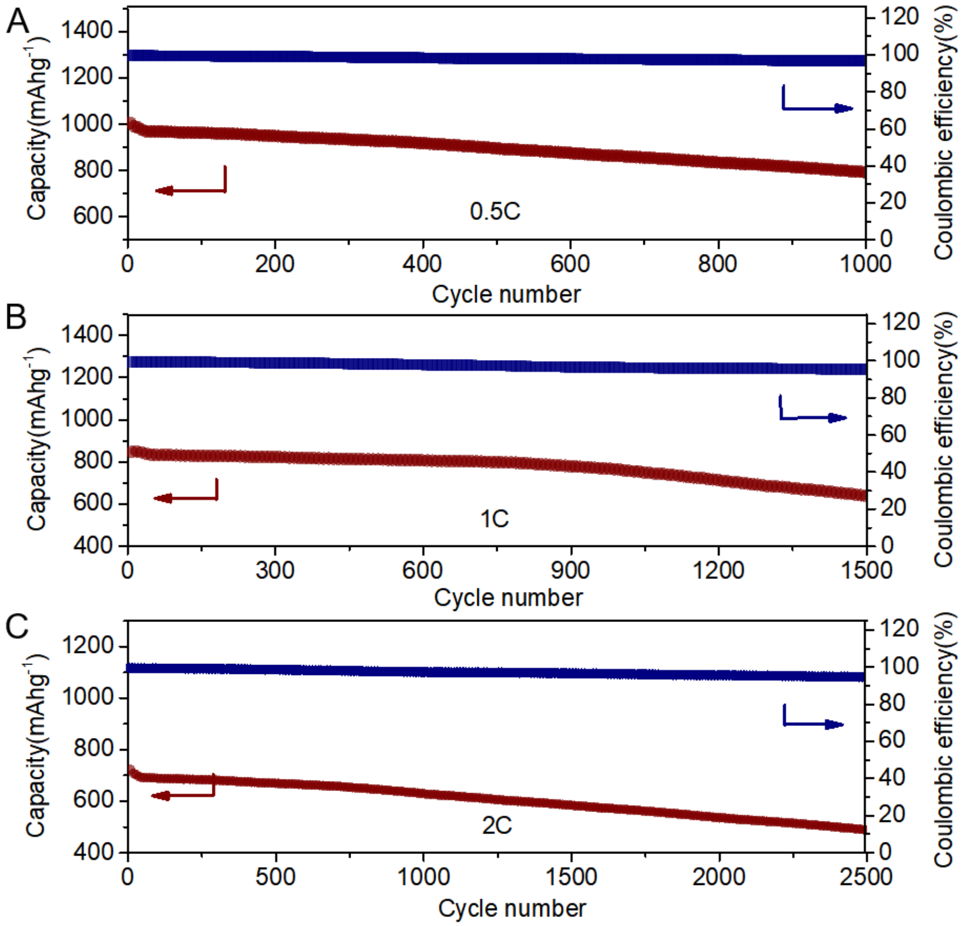

Supplementary Fig 21. Cycling performance Li-S batteries with *np-ANF* membrane (A) at a rate of 0.5 C over 1000 cycles; (B) at a rate of 1C over 1500 cycles; (C) at a rate of 2C over 2500 cycles at a sulfur loading of 1.2mg/cm^{-2}

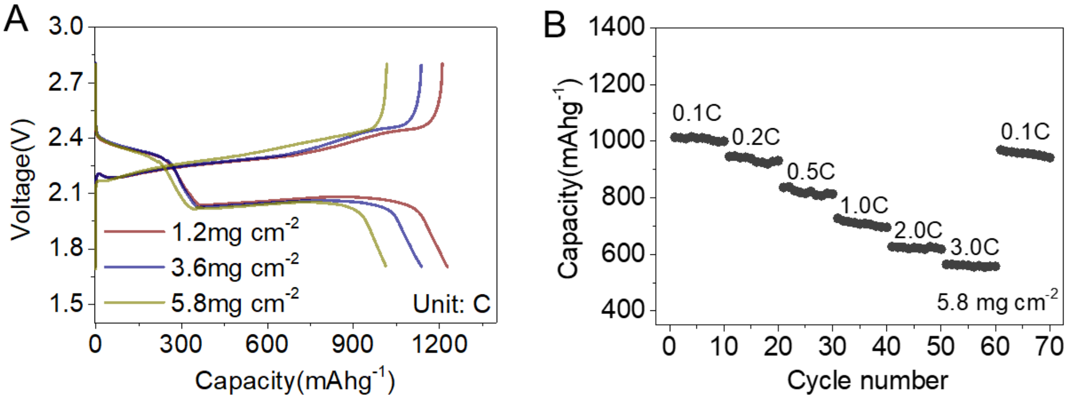

Supplementary Fig 22. Electrochemical performance of the Li-S batteries with *np-ANF* with various sulfur loadings. (A) Charge and discharge curve of Li-S batteries with *np-ANF* membrane at various sulfur loadings. (B). Rate performance of Li-S batteries ranged 0.1C to 3C with *np-ANF* at a sulfur loading of 5.8mg cm^{-2}.

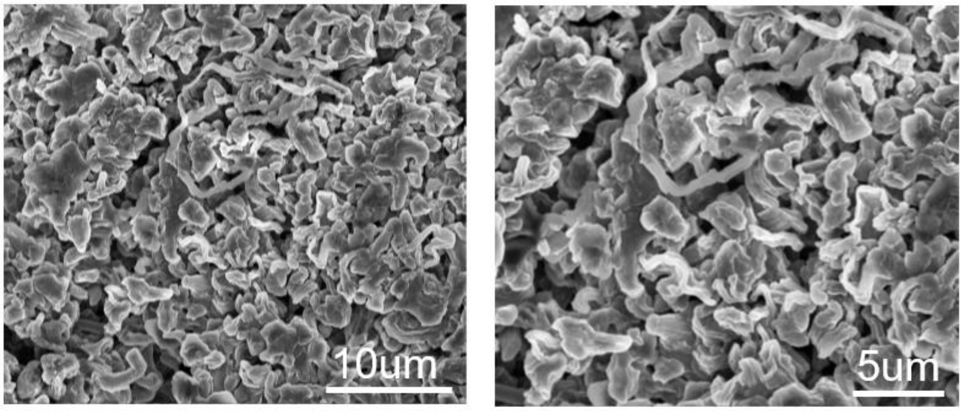

Supplementary Fig 23． SEM images of the lithium electrode with *Celgard^{TM} 2400* membrane after 250 hours cycles of stripping/plating in 1mol/L $LiCF_3SO_3$ DOL: DME v/v = 1/1) at a current density of 2mAcm^{-2}.

Supplementary Table 1**:** Tensile strength, Young's modulus, Tensile toughness and elongation at break of *np-ANF* and *Celgard^{TM} 2400* membrane

**Condition**	**Sample**	**Thickness (μm)**	**Tensile strength (MPa)**	**Young's modulus (GPa)**	**Tensile toughness (J/m^3)**	**Elongation at break (%)**
np-ANF	**Dry**	5.8±0.5	167.4±8.4	9.2±0.5	$3.0*10^3$ ±76	1.82±0.09
	**Wet**	5.8±0.5	154.5±7.7	8.1±0.4	$3.0*10^3$ ±80	1.91±0.1
	80℃	5.8±0.5	142.3±7.1	3.5±0.2	$5.9*10^3$ ±150	4.12±0.2
	After electrochemical testing	5.8±0.5	155.1±7.8	7.2±0.4	$3.3*10^3$ ±75	2.15±0.2
Celgard^{TM} 2400 longitudinal	**Dry**	25±1.3	74.5±3.7	0.17±0.02	$3.22*10^4$ ±181	43.8±2.2
	**Wet**	25±1.3	73.8±3.7	0.15±0.03	$3.68*10^4$ ±183	49.8±2.5
	80℃	25±1.3	54.4±2.7	0.05±0.003	$6.1*10^4$ ±260	113±5.7
	After electrochemical testing	25±1.3	63.7±3.2	0.11±0.07	$3.6*10^4$ ±220	56.5±6.8
Celgard^{TM} 2400 transverse	**Dry**	25±1.3	14.3±0.7	$1.28*10^{-3}$ ± $0.07*10^{-3}$	$1.47*10^5$ ±$0.07*10^5$	1033±50
	**Wet**	25±1.3	13.7±0.7	$1.27*10^{-3}$ ± $0.06*10^{-3}$±	$1.47*10^5$ ±$0.07*10^5$	1074±54
	80℃	25±1.3	6.8±0.34	$0.4*10^{-3}$ ± $0.02*10^{-3}$	$1.15*10^5$ ±$0.05*10^5$	1690±84
	After electrochemical testing	25±1.3	10.3±1.5	$0.95*10^{-3}$ ± $0.08*10^{-3}$±	$1.12*10^5$ ±$0.11*10^5$	1085±100

Supplementary Table 2**.** Young's moduli and internal resistances data for different materials for batteries.

Membrane	Description	Young's modulus (MPa)	Internal resistances	REF
np-ANF	**This work**	$9.2x10^3$	1.2	
ANFs	Aramid nanofibers membrane prepared by vacuum filtration	$8.8\ x10^3$	2.57	7
ANFs	Aramid nanofibers(20-50nm) membrane	$1x10^3$	~5	8
$(PEO/ANF)_{200}$	It stands for $(PEO/Aramid\ nanofibers)_{200}$ composite membrane	$5x10^3$	0.25	9
ANFs/PEO	It stands 15% Aramid nanofibers / Poly(ethylene oxide) composite membrane	16.5	223	10
ANFs/PSS	It stands 15% Aramid nanofibers polyphenylene sulfide composite membrane	528	1.66	11
PBO	PBO stands for Poly-p-Phenylene Benzobisoxazole memberane	$2x10^4$	2.2	12
Cellulose/PVDF-HFP	It stands for Cellulose/ Poly(vinylidene fluoride-hexafluoropropylene composite nonwoven	960	1.2	13
Cellulose/PSA	It stands for Cellulose/Polysulfonamide composite membrane	250	10	14
PE/PI	It stands for Polyethylene co-polyimide copolymer composite membrane	308	9.8	15
PVdF/PMIA/PVdF	PVdF/PMIA/PVdF stands for PVdF/ Poly(m-phenylene isophthalamide) /PVdF nanofiber composite membrane	30.3	1.5	16
PVDF-HFP/Sb_2O_3	It stands for PVDF-HFP/Sb_2O_3 composite membrane	7.52	16.13	17
PVDF	It stands for PVDF porous membrane	62.08	2.65	18
PEO/ceramic	It stands for Poly(ethylene oxide)/ceramic composite membrane	103	2631	19
PEO/POSS	It stands for PEO/polyhedral oligomeric silsesquioxane composite membrane	38.5	535	20
PMMA/Silica	It stands for Poly(methylmethacrylate) /Silica composite membrane	0.9	10.5	21
PAN gel	It stands for Polyacrylonitrile gel membrane	0.4	5.6	22
3D-GPE	It stands for Diglycidyl ether of bisphenol-A, Poly(ethylene glycol) diglycidyl ether, Diamino-poly(propylene oxide) composite gel	6.56	5	23
PET	It stands Polyethyleneterephthalate nanofiber membrane	26.6	2.5	24

Nylon 6,6/SiO_2	It stands Nylon 6,6/SiO_2 composite membrane	88	12	[25]

Supplementary Table 3. Ion-transport parameters for *np-ANF* membranes and *Celgard^{TM} 2400*

**Parameters**	***np-ANF***	***Celgard^{TM} 2400***
**Li^+** transfer number	0.63	0.68
**D_{Li+} (cm^2 s^{-1})** A (anodic peak)	$9.263 \cdot 10^{-8}$	$9.693 \cdot 10^{-8}$
**D_{Li+} (cm^2 s^{-1})** B (cathodic peak)	$5.310 \cdot 10^{-8}$	$5.129 \cdot 10^{-8}$
**D_{Li+} (cm^2 s^{-1})** C (cathodic peak)	$0.421 \cdot 10^{-8}$	$0.567 \cdot 10^{-8}$

Supplementary Table 4. Fitting results of EIS plots in Figure S18.

**Parameters**	***np-ANF***		***Celgard^{TM} 2400***	
	Before cycling	*After 100 cycles*	*Before cycling*	*After 100 cycles*
**R_0 (Ω)**	*1.2*	*7.2*	*3.9*	*11.2*
**R_{ct}//CPE_{ct} (Ω)**	*255.4*	*93.6*	*166.1*	*162.8*
**R_{sf}//CPE_{sf} (Ω)**	--	--	--	*56*

Supplementary Table 5**.** The summary of comparative performance for Li-S batteries with different structural designs of membranes.

**Functional Membrane coating on to PP/PE**	**Electrochemical Performance**					**REF**
	**Initial discharge capacity (mAhg^{-1})**	**Capacity retention (%)**	**Capacity fade per cycle(%)**	**Current density(1C = 1675 mA g^{-1})**	**Numbe r of cycle**	
np-ANF	*1268±38*	*85±2.6%*	*0.092%*	*0.1C*	*300*	
np-ANF	*969±28*	*79.2±2.4%*	*0.02%*	*0.5C*	*1000*	
np-ANF	*889±26*	*76.0±2.3%*	*0.016%*	*1.0C*	*1500*	
np-ANF	*703± 20*	*69.6±2.1%*	*0.012%*	*2.0C*	*2500*	
np-ANF	*521±16*	*64.7±1.9%*	*0.01%*	*3.0C*	*3500*	
Graphene oxide	1170	32%	0.93%	1.0C	400	[26]
Graphene oxide	920	77%	0.23%	0.1C	100	[27]
Graphene	1052	70%	0.1%	0.91C	300	[28]
SWCNT	1132	44.2%	0.19%	0.2C	300	[29]
MWCNT	1073	47%	0.14%	1.0C	300	[30]
MWCNT/PEG	1206	52%	0.16%	0.2C	300	[31]
Microporous carbon/PEG	1307	45%	0.11%	0.2C	500	[32]
Carbon nanofiber	1270	74%	0.13%	0.5C	200	[33]
Carbon	1386	60%	0.20%	0.2C	200	[34]
Carbon paper	1176	85%	0.15%	1.0C	100	[35]
Carbon black	1350	55%	0.09%	0.2C	500	[36]
N-porous carbon/PP	882	88%	0.024%	1.0C	500	[37]
N-doped-carbon nanowire	1123	60%	0.08%	0.2C	500	[38]
Cobalt/nitrogen co-doped carbon nanofibers	865	71.2%	0.06%	0.2C	500	[39]
C_3N_4	1100	66%	0.07%	1.0C	500	[40]
TiO_2/graphene	700	80%	0.01%	2.0C	1000	[41]
TiO_2/CNTs	627	57.5%	0.17%	0.5C	250	[42]
TiO_2/C_{65}	1206	50.3%	0.1%	0.5C	500	[43]

TiO_2/TiN	927	67%	0.017%	1.0C	2000	[44]
TiN	880	63.6%	0.091%	1.0C	400	[45]
NbN	815	75.8%	0.061%	1.0C	300	[46]
V_2O_5	880	91.2%	0.035%	0.0667C	250	[47]
V_2O_5/carbon nanofiber	1400	28.5%	0.07%	3.0C	1000	[48]
Al_2O_3	967	61.5%	0.77%	0.2C	50	[49]
Al_2O_3/graphene	1067.7	75%	0.25%	0.2C	100	[50]
Al_2O_3/CNT	1287	63%	0.37%	0.2C	100	[51]
Li@Nafion/PEP/Al_2O_3	924	77.2%	0.022%	1.0C	1000	[52]
SnO_2	622	68%	0.064%	0.2C	500	[53]
Co_9S_8	986	83.1%	0.011%	1.0C	1500	[54]
$Li_4Ti_5O_{12}$/graphene	813.3	85.7%	0.03%	1.0C	500	[55]
PEDOT:PSS	748	64.3%	0.0714%	1.0C	500	[56]
MoS_2	808	50%	0.083%	0.5C	600	[6]
MoS_2/CNTs	1237	52.4%	0.061	0.5C	500	[57]
MOF/CNTs	1101	50%	0.165%	0.25C	300	[58]
MOF/GO	612	71%	0.019%	1.0C	1500	[59]
MOF/SiO_2	1400	42.9%	0.57%	0.1C	100	[60]
Ce-MOFs/CNT/PP	1021	82%	0.022%	1.0C	800	[61]
$Ni_3(HITP)_2$/PP	851	84.1%	0.032%	1.0C	500	[62]
Ti_3C_2/CNTs	1240	51.6%	0.043%	0.5C	1200	[63]
MnO_2/graphene/CNTs	829	27.5%	0.029%	1.0C	2500	[64]
NiFe/N-doped graphene	812	40%	0.06%	2.0C	1000	[65]
Glass fiber	630	80%	0.04%	0.5C	500	[66]
Red phosphorus/PP	889	82%	0.036%	1.0C	500	[67]
Nafion	781	60%	0.08%	1.0C	500	[68]
Nafion/GO	1057	46%	0.18%	1.0C	300	[69]
PMIA	773.6	73%	0.045%	1.0C	600	[70]
PMIA	1121.5	63.5%	0.06%	0.5C	600	[71]
PD/PI	1404	63.4%	0.366%	0.5C	100	[72]
PAA	600	56%	0.074%	0.5C	600	[73]
PAH/PAA	1418	30%	1.4%	--	50	[74]

PAN/PMMA	1000	65%	0.175%	2.0C	200	75
PZI	940	88%	0.012%	1.0C	1000	76
Zn_2(benzimidazolate)$_2$ $(OH)_2$	1272	58%	0.2%	0.25C	200	77

Supplementary Table 6**.** Comparison of electrochemical performance of the *np-ANF* membranes with that of recent publications in Li–S batteries with various separators in the case of high sulfur loadings more than 3 mg cm^{-2}

**Separator Membrane**	**Cathode**	**Sulfur loading (mg cm^{-2})**	**Electrochemical Performance**					**REF**
			**Initial discharge capacity (mAhg^{-1})**	**Initial areal capacity (mAhcm^{-2})**	**Capacity fade per cycle (%)**	**Current density (1C = 1675 mA g^{-1})**	**Number of cycle**	
np-ANF	*C/S*	*5.8*	*1018±30*	*5.9±0.18*	*0.085± 0.003%*	*0.1 C*	*200*	
np-ANF	*C/S*	*5.8*	*945±28*	*5.5±0.16*	*0.066± 0.002%*	*0.2 C*	*500*	
MoS_2-Polymer /Celgard	C/S	4.0	~800	~3.2	0.2%	1.0 C	400	78
Co_9S_8/Celgard	C/S	5.6	985	5.5	0.079%	0.1C	200	79
V_2O_5/graphene/ Celgard	C/S	5.5	~780	4.3	~0.102%	0.1C	100	80
Red phosphorus/PP	C/S	5.0	620	3.1	0.194%	0.3C	100	67
Co/NCNS/CNT/ Celgard	C/S	5.0	1134	5.67	0.108%	1.0 C	500	81
C_3N_4/Celgard	C/S	5.0	1134	5.11	0.51%	0.1C	40	82
NbN/PP	C/S	4.0	815	3.3	0.08%	1.0C	300	46
MOF/PAN	rGO/S	7.7	1102	8.5	0.17%	0.2C	50	83
Li@Nafion/PEP/Al_2O_3	rGO/S	7.6	1087	8.26	0.114%	0.2C	300	52
TiO_2/TiN/Celgard	Graphene/S	4.3	493	2.1	0.033%	1.0C	2000	44
N-porous carbon/PP	CNT/S	6.0	977	5.8	0.047%	0.5C	400	37

Cellulose nanofiber paper	CNT/S	3.0	~830	~2.5	0.05%	0.1C	200	84
$Ni_3(HITP)_2$/PP	CNT/S	8.0	1055	8.4	0.071%	0.5C	200	62
D-HVS/PP	Carbon nanofiber/S	9.2	905	8.3	0.237%	0.2C	120	85
Ce-MOFs/CNT/PP	Carbon nanofiber/S	6.0	993.5	5.9	0.054%	0.1C	200	61
MOF/PVDF	Carbon cloth/S	5.8	1269	7.46	0.13%	0.1C	200	86

Supplementary Table 7**.** The summary of comparative performance for Li-S batteries at high temperature

**Membrane**	**Cathode material**	**Operation temperature (℃)**	**Electrochemical Performance**				**Electrolyte (LiTFSI)**	**REF**
			**Initial discharge capacity (mAhg^{-1})**	**Capacity fade per cycle (%)**	**Current density (1C = 1675 mA g^{-1})**	**Number of cycle**		
np-ANF	*C/S*	*80*	*1346±40*	*0.15%*	*0.1C*	*100*	*(DOL+DME)*	
np-ANF	*C/S*	*80*	*801±24*	*0.081%*	*3C*	*500*	*(DOL+DME)*	
C@PI@LLZO	C/S	80	897.1	0.2%	5C	200	(DOL+DME)	87
CuNWs-GN/PI/LLZO	C/S	80	817.8	0.24%	0.5Ag^{-1}	50	(DOL+DME)	88
PAN@APP	C/S	75	~700	0.220%	1.0C	100	(DOL+DME)	89
Celgard™ 2325	Carbon nanotube/S	70	~750	~0.444%	2.0C	150	(DOL+DME)	90
Celgard™ 2400	Graphene/BN-S	70	1032	0.047%	2.0C	300	(DOL+DME)	91
PE	S@pPAN	60	820.3	--	0.2C	200	(TEP+TTE)	92
Li@Nafion/PE/Al_2O_3	RGO@S	60	1172	0.059%	0.2C	500	(DOL+DME)	52
Celgard™ 2400	Porous graphene/S	60	~590	0.297%	1.0C	80	(DOL+DME)	93

Celgard™ 2400	Alucone coated C/S	55	1065	0.152%	0.1C	300	$LiPF_6$ (EC:DEC:EMC)	94
Celgard™ 2400	S@CNTs/ $Co_3S_4NB_s$	50	953	0.082%	0.2C	300	(DOL+DME)	95
CNT-COF	C/S	50	450	0.16%	$2.0Ag^{-1}$	300	(DOL+DME)	96
Celgard™ 2400	Mesoporous C/S	45	~1180	~4.067%	0.1C	10	(DOL:DME:BTFE)	97
Tonen polyolefin	C/S	45	-	0.770%	0.1C	50	(DOL+DME)	98

Supplementary Table 8**.** Multiparameter comparison with various modified separator for Li-S batteries in the form of glyph plots.

	**Discharge Capacity 0.1C ($mAhg^{-1}$)**	**Discharge Capacity 1C ($mAhg^{-1}$)**	**Cycle number**	**Capacity Retention 1C (%)**	**CE (%)**	**Sulfur loading (mg/cm^2)**	**Operational Temperature (℃)**	**REF**
This work	*1268±38*	*889±26*	*1500*	*76*	*98*	*5.8*	*80*	
NANF/Celgard	*1270*	*760*	*800*	*62.4*	*98*	*5.2*	*70*	99
PMIA/MOF	*1391*	*901*	*350*	*88*	*96.9*	*9.23*	*80*	100
GO/Celgard	1403	1100	400	32	98	1.1	22	26
N-porous carbon/PP	1257	851	500	88	98	6.0	22	37
MWCNT/Celgard	1324	1073	300	47	99	1.2	22	30
Carbon paper/Celgard	1367	1176	100	85	96	1.2	22	35
MoS_2/Celgard	1300	1007	2000	42	98	4.0	22	78
Red phosphorus/PP	1200	889	500	82	99	5.0	22	67
Co_9S_8/Celgard	1360	986	1500	83	98	5.6	22	54
$Ni_3(HITP)_2$/PP	1186	879	500	84	99	8.0	22	62
MOF@GO	1072	612	1500	71	98	0.7	22	59
MnO_2/graphene/CNTs	1259	960	2500	27.5	99	2.37	22	64
PAN@APP	1310	815	400	77.8	99	1.8	75	89
Ce-MOFs/CNT/PP	1200	1021	800	82	99	6.0	22	61

Li@Nafion/PEP/Al_2O_3	1398	924	1000	77.2	99	7.6	60	[52]
PZI	1095	940	1000	85	99	5.8	25	[76]
$Li_4Ti_5O_{12}$/graphene/Celgard	1408	813	500	85	98	1.1	22	[55]
PEDOT:PSS	914	748	500	64.3	99	2.9	22	[56]
PMIA/Celgard	944	773	600	73	98	0.6	22	[70]
TiO_2/TiN/Celgard	1250	790	2000	85	99	4.3	22	[44]
NbN/Celgard	1400	815	300	81.7	99	4.0	22	[46]
Nafion/Celgard	960	718	500	60	98	0.53	22	[68]
C_3N_4/Celgard	1200	1100	500	66	99	5.0	22	[40]

SUPPLEMENTARY REFERENCES

1. Picart, C. *et al.* Molecular basis for the explanation of the exponential growth of polyelectrolyte multilayers. *Proc. Natl. Acad. Sci. U. S. A.* **99**, 12531–5 (2002).
2. Porcel, C. *et al.* Influence of the polyelectrolyte molecular weight on exponentially growing multilayer films in the linear regime. *Langmuir* **23**, 1898–904 (2007).
3. Zhu, J., Watts, D. & Kotov, N. A. Gelation-Assisted Layer-by-Layer Deposition of High Performance Nanocomposites. *Zeitschrift für Phys. Chemie* **232**, 1383–1398 (2018).
4. Chen, Y. *et al.* Electrospun PMIA and PVDF-HFP composite nanofibrous membranes with two different structures for improved lithium-ion battery separators. *Solid State Ionics* **347**, 115253 (2020).
5. Sun, J. *et al.* Entrapment of Polysulfides by a Black-Phosphorus-Modified Separator for Lithium–Sulfur Batteries. *Adv. Mater.* **28**, 9797–9803 (2016).
6. Ghazi, Z. A. *et al.* MoS2/Celgard separator as efficient polysulfide barrier for long-life lithium–sulfur batteries. *Adv. Mater.* **29**, 1606817 (2017).
7. Patel, A. *et al.* High Modulus, Thermally Stable , and Self-Extinguishing Aramid Nanofiber Separators. *ACS Appl. Mater. Interfaces* **12**, 25756–25766 (2020).
8. Li, J., Tian, W., Yan, H., He, L. & Tuo, X. Preparation and performance of aramid nanofiber membrane for separator of lithium ion battery. *J. Appl. Polym. Sci.* **133**, 43623 (2016).
9. Tung, S. O., Ho, S., Yang, M., Zhang, R. & Kotov, N. A. A dendrite-suppressing composite ion conductor from aramid nanofibres. *Nat. Commun.* **6**, 6152 (2015).
10. Liu, L. *et al.* membranes with 3D aramid nanofiber frameworks for stable all-solid-state lithium metal batteries. *Sci. China Mater.* **63**, 703–718 (2020).

11. Zhu, C. *et al.* Aramid nanofibers/ polyphenylene sulfide nonwoven composite separator fabricated through a facile papermaking method for lithium ion battery. *J. Memb. Sci.* **588**, 117169 (2019).

12. Hao, X. *et al.* Ultrastrong polyoxyzole nanofiber membranes for dendrite-proof and Heat-resistant battery separators. *Nano Lett.* **16**, 2981–2987 (2016).

13. Zhang, J. *et al.* Renewable and superior thermal-resistant cellulose-based composite nonwoven as lithium-ion battery separator. *ACS Appl. Mater. Interfaces* **5**, 128–134 (2013).

14. Xu, Q. *et al.* Cellulose/Polysulfonamide Composite Membrane as a High Performance Lithium-Ion Battery Separator. *ACS Sustain. Chem. Eng.* **2**, 194–199 (2014).

15. Song, J. *et al.* Co-polyimide-coated polyethylene separators for enhanced thermal stability of lithium ion batteries. *Electrochim. Acta* **85**, 524–530 (2012).

16. Zhai, Y. *et al.* Sandwich-structured PVdF/PMIA/PVdF nanofibrous separators with robust mechanical strength and thermal stability for lithium ion batteries. *J. Mater. Chem. A* **2**, 14511 (2014).

17. Ansari, Y. *et al.* Low-cost, dendrite-blocking polymer-Sb2O3 separators for lithium and sodium batteries. *J. Electrochem. Soc.* **161**, A1655–A1661 (2014).

18. Ji, G. L., Zhu, B. K., Cui, Z. Y., Zhang, C. F. & Xu, Y. Y. PVDF porous matrix with controlled microstructure prepared by TIPS process as polymer electrolyte for lithium ion battery. *Polymer (Guildf).* **48**, 6415–6425 (2007).

19. Leo, C. J., Subba Rao, G. V. & Chowdari, B. V. R. Studies on plasticized PEO-lithium triflate-ceramic filler composite electrolyte system. *Solid State Ionics* **148**, 159–171 (2002).

20. Pan, Q., Smith, D. M., Qi, H., Wang, S. & Li, C. Y. Hybrid Electrolytes with Controlled Network Structures for Lithium Metal Batteries. *Adv. Mater.* **27**, 5995–6001 (2015).

21. Gayet, F. *et al.* Unique combination of mechanical strength, thermal stability, and high ion conduction in PMMA - Silica nanocomposites containing high loadings of ionic liquid. *Chem. Mater.* **21**, 5575–5577 (2009).

22. Patel, M., Chandrappa, K. G. & Bhattacharyya, A. J. Increasing ionic conductivity and mechanical strength of a plastic electrolyte by inclusion of a polymer. *Electrochim. Acta* **54**, 209–215 (2008).

23. Lu, Q. *et al.* Dendrite-free, high-rate, long-life lithium metal batteries with a 3D cross-linked network polymer electrolyte. *Adv. Mater.* **29**, 1604460 (2017).

24. Hao, J. *et al.* A novel polyethylene terephthalate nonwoven separator based on electrospinning technique for lithium ion battery. *J. Memb. Sci.* **428**, 11–16 (2013).

25. Yanilmaz, M., Dirican, M. & Zhang, X. Evaluation of electrospun SiO2/nylon 6,6 nanofiber membranes as a thermally-stable separator for lithium-ion batteries. *Electrochim. Acta* **133**, 501–508 (2014).

26. Shaibani, M. *et al.* Suppressed Polysulfide Crossover in Li-S Batteries through a High-Flux Graphene Oxide Membrane Supported on a Sulfur Cathode. *ACS Nano* **10**, 7768–7779 (2016).

27. Huang, J.-Q. *et al.* Permselective Graphene Oxide Membrane for Highly Stable and Anti-Self-Dischage Lithium-Sulfur Batteries. *ACS Nano* **9**, 3002–3011 (2015).

28. Li, F. *et al.* A graphene-pure-sulfur sandwich structure for ultrafast, long-life lithium-sulfur batteries. *Adv. Mater.* **26**, 625–631 (2014).

29. Chang, C. H., Chung, S. H. & Manthiram, A. Effective Stabilization of a High-Loading Sulfur Cathode and a Lithium-Metal Anode in Li-S Batteries Utilizing SWCNT-Modulated Separators. *Small* **12**, 174–179 (2016).

30. Chung, S. H. & Manthiram, A. High-performance Li-S batteries with an ultra-lightweight MWCNT-coated separator. *J. Phys. Chem. Lett.* **5**, 1978–1983 (2014).

31. Luo, L., Chung, S. H. & Manthiram, A. A trifunctional multi-walled carbon nanotubes/polyethylene glycol (MWCNT/PEG)-coated separator through a layer-by-layer coating strategy for high-energy Li-S batteries. *J. Mater. Chem. A* **4**, 16805–16811 (2016).

32. Chung, S. H. & Manthiram, A. A polyethylene glycol-supported microporous carbon coating as a polysulfide trap for utilizing pure sulfur cathodes in lithium-sulfur batteries. *Adv. Mater.* **26**, 7352–7357 (2014).

33. Chung, S. H., Han, P., Singhal, R., Kalra, V. & Manthiram, A. Electrochemically Stable Rechargeable Lithium-Sulfur Batteries with a Microporous Carbon Nanofiber Filter for Polysulfide. *Adv. Energy Mater.* **5**, 1–12 (2015).

34. Chung, S. H. & Manthiram, A. Bifunctional separator with a light-weight carbon-coating for dynamically and statically stable lithium-sulfur batteries. *Adv. Funct. Mater.* **24**, 5299–5306 (2014).

35. Su, Y. S. & Manthiram, A. Lithium-sulphur batteries with a microporous carbon paper as a bifunctional interlayer. *Nat. Commun.* **3**, 1166 (2012).

36. Yao, H. *et al.* Improved lithium–sulfur batteries with a conductive coating on the separator to prevent the accumulation of inactive S-related species at the cathode–separator interface. *Energy Environ. Sci.* **7**, 3381–3390 (2014).

37. Pei, F. *et al.* A Two-dimensional porous carbon-modified separator for high-energy-density Li-S batteries. *Joule* **2**, 323–336 (2018).

38. Zhou, X. *et al.* A high-level N-doped porous carbon nanowire modified separator for long-life lithium-sulfur batteries. *J. Electroanal. Chem.* **768**, 55–61 (2016).

39. Chen, G. *et al.* A multifunctional separator modified with cobalt and nitrogen co-doped porous carbon nanofibers for Li–S batteries. *J. Memb. Sci.* **548**, 247–253 (2018).

40. Fan, C. Y. *et al.* The Effective Design of a Polysulfide-Trapped Separator at the Molecular Level for High Energy Density Li-S Batteries. *ACS Appl. Mater. Interfaces* **8**, 16108–16115 (2016).

41. Xiao, Z. *et al.* A lightweight TiO2/Graphene interlayer, applied as a highly effective polysulfide absorbent for fast, long-life lithium-sulfur batteries. *Adv. Mater.* **27**, 2891–2898 (2015).

42. Xu, G. *et al.* Absorption mechanism of carbon-nanotube paper-titanium dioxide as a multifunctional barrier material for lithium-sulfur batteries. *Nano Res.* **8**, 3066–3074 (2015).

43. Xu, G. *et al.* A thin multifunctional coating on a separator improves the cyclability and safety of lithium sulfur batteries. *Chem. Sci.* **8**, 6619–6625 (2017).

44. Zhou, T. *et al.* Twinborn TiO2-TiN heterostructures enabling smooth trapping-diffusion-conversion of polysulfides towards ultralong life lithium-sulfur batteries. *Energy Environ. Sci.* **10**, 1694–1703 (2017).

45. Qi, B. *et al.* Mesoporous TiN microspheres as an efficient polysulfide barrier for lithium–sulfur batteries. *J. Mater. Chem. A* (2018). doi:10.1039/C8TA04920C

46. Kim, S. *et al.* Simultaneous suppression of shuttle effect and lithium dendrite growth by lightweight bifunctional separator for Li-S batteries. *ACS Appled Energy Mater.* **3**, 2643–2652 (2020).

47. Li, W. *et al.* V2O5polysulfide anion barrier for long-lived Li-S batteries. *Chem. Mater.* **26**, 3404–3410 (2014).

48. Liu, M. *et al.* Suppressing Self-Discharge and Shuttle Effect of Lithium–Sulfur Batteries with V2O5-Decorated Carbon Nanofiber Interlayer. *Small* **13**, 1–7 (2017).

49. Zhang, Z., Lai, Y., Zhang, Z., Zhang, K. & Li, J. Al2O3-coated porous separator for enhanced electrochemical performance of lithium sulfur batteries. *Electrochim. Acta* **129**, 55–61 (2014).

50. Song, R. *et al.* A trilayer separator with dual function for high performance lithium-sulfur batteries. *J. Power Sources* **301**, 179–186 (2016).

51. Xu, Q., Hu, G. C., Bi, H. L. & Xiang, H. F. A trilayer carbon nanotube/Al2O3/polypropylene separator for lithium-sulfur batteries. *Ionics (Kiel).* **21**, 981–986 (2015).

52. He, Y., Wu, S., Li, Q. & Zhou, H. Designing a multifunctional separator for high-performance Li-S batteries at elevated temperature. *Small* **15**, 1904332 (2019).

53. Xiang, Y. *et al.* Interfacing soluble polysulfides with a SnO2 functionalized separator: An efficient approach for improving performance of Li-S battery. *J. Memb. Sci.* **563**, 380–387 (2018).

54. Science, E. Vertical Co9S8 hollow nanowall arrays grown on Celgard separator as a multifunctional polysulfide barrier for high- performance Li-S batteries. (2018). doi:10.1039/x0xx00000x

55. Zhao, Y. *et al.* Dense coating of Li4Ti5O12 and graphene mixture on the separator to produce long cycle life of lithium-sulfur battery. *Nano Energy* **30**, 1–8 (2016).

56. Abbas, S. A. *et al.* Bifunctional separator as a polysulfide mediator for highly stable Li-S batteries. *J. Mater. Chem. A* **4**, 9661–9669 (2016).

57. Yan, L. *et al.* Enhanced performance of lithium-sulfur batteries with an ultrathin and lightweight MoS2/carbon nanotube interlayer. *J. Power Sources* **389**, 169–177 (2018).

58. Li, M. *et al.* Metal-Organic Framework-Based Separators for Enhancing Li-S Battery Stability: Mechanism of Mitigating Polysulfide Diffusion. *ACS Energy Lett.* **2**, 2362–2367 (2017).

59. Bai, S., Liu, X., Zhu, K., Wu, S. & Zhou, H. Metal-organic framework-based separator for lithium-sulfur batteries. *Nat. Energy* **1**, (2016).

60. Suriyakumar, S., Stephan, A. M., Angulakshmi, N., Hassan, M. H. & Alkordi, M. H. Metal–organic framework@SiO $_2$ as permselective separator for lithium–sulfur batteries. *J. Mater. Chem. A* (2018). doi:10.1039/C8TA02259C

61. Hong, X. *et al.* Cerium based metal-organic frameworks as an efficient separator coating catalyzing the conversion of polysulfi des for high performance lithium-sulfur batteries. *ACS Nano* **13**, 1923–1931 (2019).

62. Zang, Y. *et al.* Large-Area Preparation of Crack-Free Crystalline Microporous Conductive Membrane to Upgrade High Energy Lithium – Sulfur Batteries. *Adv. Energy Mater.* **8**, 1802052 (2018).

63. Liang, X., Rangom, Y., Kwok, C. Y., Pang, Q. & Nazar, L. F. Interwoven MXene

Nanosheet/Carbon-Nanotube Composites as Li–S Cathode Hosts. *Adv. Mater.* **29**, 1–7 (2017).

64. Kong, W. *et al.* Ultrathin MnO2/Graphene Oxide/Carbon Nanotube Interlayer as Efficient Polysulfide-Trapping Shield for High-Performance Li–S Batteries. *Adv. Funct. Mater.* **27**, (2017).

65. Peng, H. J. *et al.* A Cooperative Interface for Highly Efficient Lithium–Sulfur Batteries. *Adv. Mater.* **28**, 9551–9558 (2016).

66. Wang, L., Liu, J., Haller, S., Wang, Y. & Xia, Y. A scalable hybrid separator for a high performance lithium–sulfur battery. *Chem. Commun.* **51**, 6996–6999 (2015).

67. Wang, Z. *et al.* Constructing metal-free and cost-effective multifunctional separator for high-performance lithium-sulfur batteries. *Nano Energy* **59**, 390–398 (2019).

68. Huang, J.-Q. *et al.* Ionic shield for polysulfides towards highly-stable lithium–sulfur batteries. *Energy Environ. Sci.* **7**, 347–353 (2014).

69. Zhuang, T. Z. *et al.* Rational Integration of Polypropylene/Graphene Oxide/Nafion as Ternary-Layered Separator to Retard the Shuttle of Polysulfides for Lithium-Sulfur Batteries. *Small* **12**, 381–389 (2016).

70. Sun, C. *et al.* Integrating flexible PMIA separator and electrode for dealing with multi-aspect issues in Li-S batteries. *Electrochim. Acta* **359**, 136987 (2020).

71. Deng, N. *et al.* Designing of a phosphorus, nitrogen, and sulfur three-flame retardant applied in a gel poly-m- phenyleneisophthalamide nanofiber membrane for advanced safety lithium- sulfur batteries. *ACS Appl. Mater. interfaces* **11**, 36705–36716 (2019).

72. Wang, Y., Zhang, Z., Dong, L. & Jin, Y. Reduced shuttle effect by dual synergism of lithium – sulfur batteries with polydopamine-modified polyimide separators. *J. Memb. Sci.* **595**, 117581 (2020).

73. Song, S., Shi, L., Lu, S. & Pang, Y. Author ' s Accepted Manuscript A new polysulfide blocker - Poly (acrylic acid) modified separator for improved performance of lithium-sulfur battery. *J. Memb. Sci.* **563**, 277–283 (2018).

74. Gu, M. *et al.* Inhibiting the shuttle effect in lithium-sulfur batteries using a layer-by-layer assembled ion-permselective separator. *RSC Adv.* **4**, 46940–46946 (2014).

75. Li, Y. *et al.* Glass fiber separator coated by porous carbon nanofiber derived from immiscible PAN/PMMA for high-performance lithium-sulfur batteries. *J. Memb. Sci.* **552**, 31–42 (2018).

76. Li, G. *et al.* Polysulfide Regulation by the Zwitterionic Barrier toward Durable Lithium-Sulfur Batteries. *JACS* **142**, 3583–3592 (2020).

77. Huang, J. K. *et al.* Functional Two-Dimensional Coordination Polymeric Layer as a Charge Barrier in Li-S Batteries. *ACS Nano* **12**, 836–843 (2018).

78. Wu, J. *et al.* Ultralight layer-by-layer self-assembled MoS2-polymer modified separator for simultaneously trapping polysulfides and suppressing lithium dendrites. *Adv. Energy Mater.* **8**, 1802430 (2018).

79. Li, S., He, J. & Chen, Y. Vertical Co9S8 hollow nanowall arrays grown on a Celgard separator as a multifunctional polysulfide. *Energy Environ. Sci.* **11**, 2560–2568 (2018).

80. Zhang, Y. & Wu, H. Interwoven V2O5 nanowire/graphene nanoscroll hybrid assembled as efficient polysulfide-trapping-conversion interlayer for long-life lithium–sulfur batteries. *J. Mater.*

Chem. A **6**, 19358–19370 (2018).

81. Song, C. *et al.* 3D catalytic MOF-based nanocomposite as separator coatings for high-performance Li-S battery. *Chem. Eng. J.* **381**, 122701 (2020).

82. Fan, C. *et al.* The effective design of a polysulfide-trapped separator at the molecular level for high energy density Li-S batteries. *ACS Appl. Mater. Interfaces* **8**, 16108–16115 (2016).

83. Zhou, C. *et al.* A robust electrospun separator modi fi ed with in situ grown metal-organic frameworks for lithium-sulfur batteries. *Chem. Eng. J.* **395**, 124979 (2020).

84. Lee, S. & Lee, S. Nanomat Li–S batteries based on all-fibrous cathode/separator assemblies and reinforced Li metal anodes: towards ultrahigh energy density and flexibility. *Energy Environ. Sci.* **12**, 177–186 (2019).

85. Wang, J. *et al.* Suppressing the shuttle effect and dendrite growth in lithium -sulfur batteries. *ACS Nano* **14**, 9819–9831 (2020).

86. He, Y. *et al.* Simultaneously inhibiting lithium dendrites growth and polysulfides shuttle by a flexible MOF-based membrane in Li-S batteries. *Adv. Energy Mater.* **8**, 1802130 (2018).

87. Zhou, Z. *et al.* Functionalized polyimide separators enable high performance lithium sulfur batteries at elevated temperature. *J. Power Sources* **396**, 542–550 (2018).

88. Zhou, Z. *et al.* A multifunctional separator enables safe and durable lithium/magnesium-sulfur batteries under elevated temperature. *Adv. Energy Mater.* **10**, 1902023 (2020).

89. Lei, T. *et al.* A nonflammable and thermotolerant separator suppresses pPolysulfide dissolution for safe and long-cycle lithium-sulfur batteries. *Adv. Energy Mater.* 1802441 (2018). doi:10.1002/aenm.201802441

90. Kim, H., Lee, J. T. & Yushin, G. High temperature stabilization of lithium e sulfur cells with carbon nanotube current collector. *J. Power Sources* **226**, 256–265 (2013).

91. Deng, D. R. *et al.* Enhanced adsorptions to polysulfides on graphene-supported BN nanosheets with excellent Li-S battery performance in a wide temperature range. *ACS Nano* **12**, 11120–11129 (2018).

92. Yang, H. *et al.* An intrinsic flame-retardant organic electrolyte for safe lithium-sulfur batteries. *Angew. Chemie Int. Ed.* **131**, 801–805 (2019).

93. Huang, J., Liu, X. & Zhang, Q. Entrapment of sulfur in hierarchical porous graphene for lithium-sulfur batteries with high rate performance from -40 to 60C. *Nano Energy* **2**, 314–321 (2013).

94. Li, X. *et al.* Safe and durable high-temperature lithium-sulfur batteries via molecular layer deposited coating. *Nano Lett.* **16**, 3545–3549 (2016).

95. Chen, T. *et al.* Self-templated formation of interlaced carbon nanotubes threaded hollow Co3S4 nanoboxes for high-rate and heat-resistant lithium-sulfur batteries. *JACS* **139**, 12710–12715 (2017).

96. Wang, J., Qin, W., Zhu, X. & Teng, Y. Covalent organic frameworks (COF)/CNT nanocomposite for high performance and wide operating temperature lithium-sulfur batteries. *Energy* doi.org/10.1016/j.energy.2020.117372 (2020). doi:10.1016/j.energy.2020.117372

97. Gordin, M. L. *et al.* Bis(2,2,2-trifluoroethyl) ether as an electrolyte co-solvent for mitigating self-discharge in lithium-sulfur batteries. *ACS Appl. Mater. Interface* **6**, 8006–8010 (2014).

98. Rolf, M. *et al.* Systematical electrochemical study on the parasitic shuttle-effect in lithium-sulfur-cells at different temperatures and different rates. *J. Power Sources* **259**, 289–299 (2014).

99. Zheng, S., Zhang, H., Fan, J., Xu, Q. & Min, Y. Improving Electrochemical Performance and Safety of Lithium-Sulfur Batteries by a “ Bulletproof Vest ”. *ACS Appl. Mater. Interfaces* **12**, 51904–51916 (2020).

100. Liu, J. *et al.* A high-safety and multifunctional MOFs modified aramid nanofiber separator for lithium-sulfur batteries. *Chem. Eng. J.* **411**, 128540 (2021).
