# Supplementary material for: Multifactorial engineering of biomimetic membranes for batteries with multiple high-performance parameters
Source: Nat Commun. 2022 Jan 12;13:278. doi: 10.1038/s41467-021-27861-w (PMC8755825; doi:10.1038/s41467-021-27861-w)
Supplement: Supplementary file 2 — Description of Additional Supplementary Files [file 41467_2021_27861_MOESM2_ESM.docx]

**Description of Additional Supplementary Files**

Multifactorial engineering of biomimetic membranes for batteries with multiple high-performance parameters

Mingqiang Wang^1,2,3,ϕ^, Ahmet Emre^2,3,4,5,ϕ^, Ji-Young Kim^2,3,4^, Yiting Huang^1^, Li Liu^1^, Volkan Cecen^2,3^, Yudong Huang^1^, Nicholas A. Kotov^2,3,4,5,^*

^1^ School of Chemistry and Chemical Engineering, Harbin Institute of Technology, Harbin 150001, P. R. China;

^2^ Department of Chemical Engineering, University of Michigan, Ann Arbor, Michigan 48109, USA;

^3^ Biointerfaces Institute, University of Michigan, Ann Arbor, Michigan 48109, USA;

^4^ Department of Materials Science and Engineering, University of Michigan, Ann Arbor, Michigan 48109, USA;

^5^ Department of Biomedical Engineering, University of Michigan, Ann Arbor, Michigan 48109, USA;

^ϕ^ These authors contributed equally: Mingqiang Wang, Ahmet Emre.

*Correspondence: kotov@umich.edu

File Name: Movie S1.

Description: Li-ion transport through nanochannel without surface charge density

File Name: Movie S2.

Description: LPS transport through nanochannel without surface charge density

File Name: Movie S3.

Description: Li-ion transport through nanochannel with surface charge density

File Name: Movie S4.

Description: Inhibition of LPS transport through nanochannel with surface charge density
